# The use of tyrosinases in a chemoenzymatic cascade as a peptide ligation strategy†

**DOI:** 10.1039/d2cb00237j

**Published:** 2022-12-12

**Authors:** Yeke Ni, Yu Wang, Alethea B. Tabor, John M. Ward, Helen C. Hailes

**Affiliations:** a Department of Chemistry, University College London 20 Gordon Street London WC1H 0AJ UK h.c.hailes@ucl.ac.uk; b Department of Biochemical Engineering, University College London Bernard Katz Building, Gower Street London WC1E 6BT UK

## Abstract

Peptides play many key roles in biological systems and numerous methods have been developed to generate both natural and unnatural peptides. However, straightforward, reliable coupling methods that can be achieved under mild reactions conditions are still sought after. In this work, a new N-terminal tyrosine-containing peptide ligation method with aldehydes, utilising a Pictet–Spengler reaction is described. In a key step, tyrosinase enzymes have been used to convert l-tyrosine to l-3,4-dihydroxyphenyl alanine (l-DOPA) residues, generating suitable functionality for the Pictet–Spengler coupling. This new chemoenzymatic coupling strategy can be used for fluorescent-tagging and peptide ligation purposes.

## Abstract 2

A new N-terminal tyrosine-containing peptide ligation method has been developed utilising tyrosinase enzymes and a subsequent Pictet-Spengler reaction with aldehydes.

## Graphical abstract

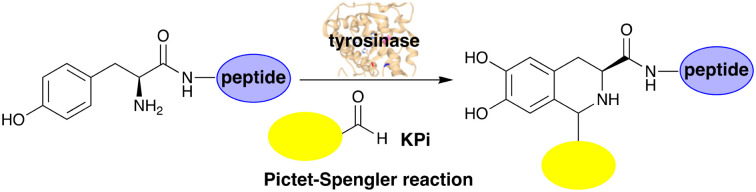

## Introduction

Enzymes have significant potential for a wide range of applications as catalysts in chemical synthesis. Enzyme catalysed reactions have many desirable features such as high yields, reaction rates, and stereoselectivities, together with a good sustainability profile.^1–4^ Protein engineering, especially enzyme mutagenesis, has also endowed biocatalysts with higher efficiencies and broader substrate acceptance for use in natural and unnatural product syntheses, enabling chemoenzymatic strategies to be more widely adopted.^4–6^

**Scheme 1:**
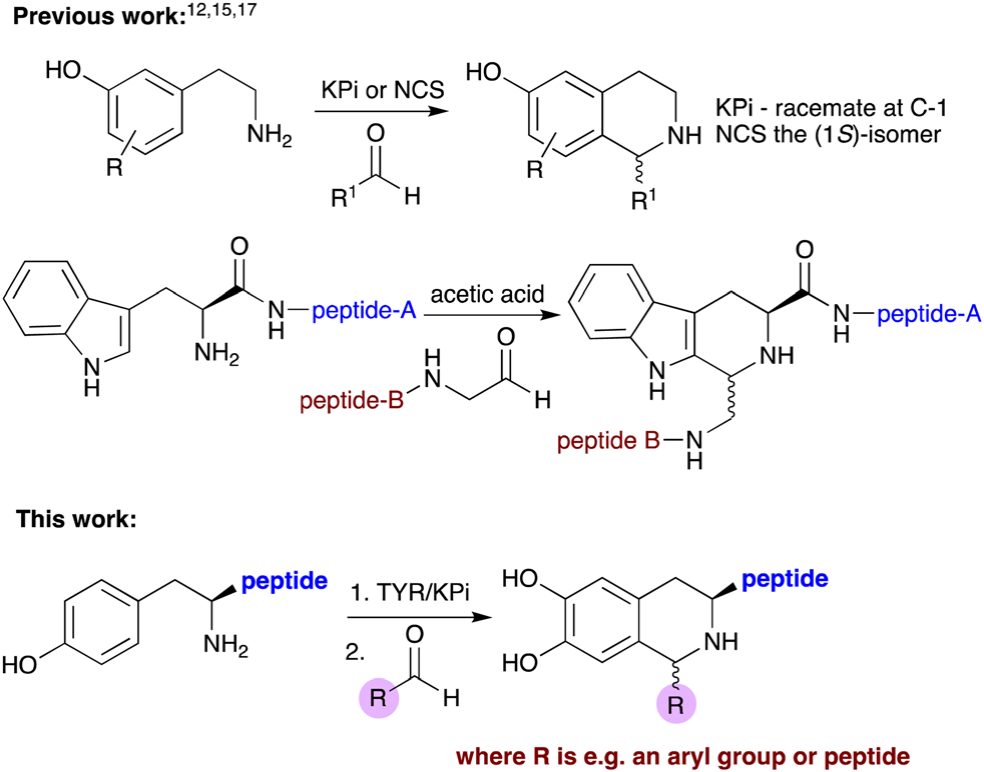
Previous synthesis of THIAs and peptide-tetrahydro-β-carbolines using PSRs and this work using TYRs in a two-step chemoenzymatic cascade.

Tyrosinases (TYRs) are Cu-dependent enzymes that convert L-tyrosine to melanin *via* oxidation of the monophenol to l-DOPA and then further oxidation. They are found widely in fungi, plants and animals and the catalytic mechanism has been well-studied,^7^ as well as its importance in food, pharmaceutical and industrial applications.^8,9^ Recently, they have been used for the selective hydroxylation of phenols in synthesis.^10,11^ For example, Wang *et al.* developed novel *in vitro* cascades with TYRs, decarboxylases and transaminases, to prepare amines and aldehydes from tyrosine and analogues, followed by a norcoclaurine synthase (NCS) enzyme-mediated Pictet–Spengler reaction (PSR) to generate unnatural tetrahydroisoquinoline alkaloids (THIAs).^11^ PSRs are a useful method to synthesize THIAs and tetrahydro-β-carboline alkaloids *via* non-enzymatic methods, for example using potassium phosphate (KPi) buffer, or enzymatic processes, the later producing products in high enantiomeric excess (ee) (Scheme 1).^11–16^ Interestingly, PSRs have been incorporated into chemical peptide ligation strategies using N-terminal tryptophan-peptides and aldehyde-tagged peptides to give coupled products with a tetrahydro-β-carboline scaffold.^17–20^ The electron-rich indole ring in tryptophan enabled these reactions to proceed under acidic conditions or aqueous buffer at 37 °C.

N- and C-terminal tyrosine-containing peptides play important roles as neurotransmitters, hormones, peptide antigens and essential signalling processes.^21–25^ Many efforts have therefore been made to modify such peptides, including selective amide formation, oxidative couplings and bioconjugation with functional proteins or labelled species, with a view to studying protein structure and exploring new approaches in diagnostics, drug design and to trigger an immune response.^10,26,27^ In this work we have investigated a chemoenzymatic method for the modification of N-terminal tyrosine residues using TYRs, opening up the potential for use as a peptide coupling or labelling strategy (Scheme 1).

## Results and discussion

### Establishing a chemoenzymatic cascade with tyrosinases

**Scheme 2:**
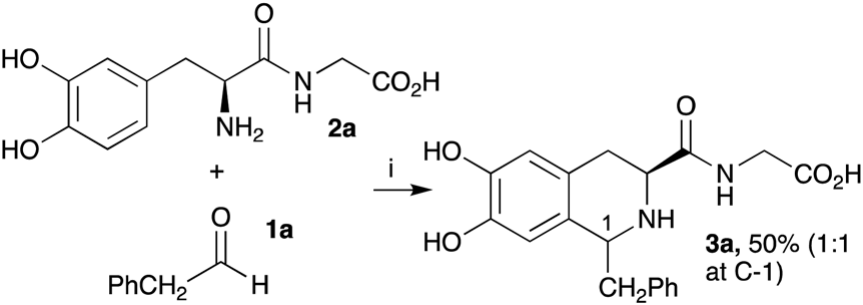
The initial PSR using DOPA-Gly 2a. Reaction conditions: (i) 2a (1 equiv.), 1a (1.5 equiv.), sodium ascorbate (2 equiv.), in 0.2 M KPi buffer pH 6/CH_3_CN (1 : 1), 50 °C, 18 h. Yields were determined by analytical HPLC (against product standards). Diastereoselectivities were determined by HPLC and ^1^H NMR spectroscopy.

Previously, Pesnot *et al.* reported the PSR between l-DOPA and phenylacetaldehyde 1a in KPi buffer to generate the corresponding THIA in good yield (65%, diastereomeric ratio (dr) 1 : 1.2).^12^ To develop procedures for coupling DOPA-peptides, generated using TYRs, initial studies investigated the ease of performing PSRs with an N-terminal DOPA-dipeptide to establish the reaction conditions (Scheme 2). l-DOPA-Gly 2a was prepared as previously described^28^ and reacted with 1a. To avoid over-oxidation of the DOPA residues to quinones, two equivalents of sodium ascorbate were added, and reactions conducted at 50 °C for 18 h, based on previously reported conditions.^12^ THIA 3a was readily formed in 50% yield as a mixture of diastereoisomers ((1*R*,3*S*) : (1*S*,3*S*) ∼1 : 1).

**Fig. 1:**
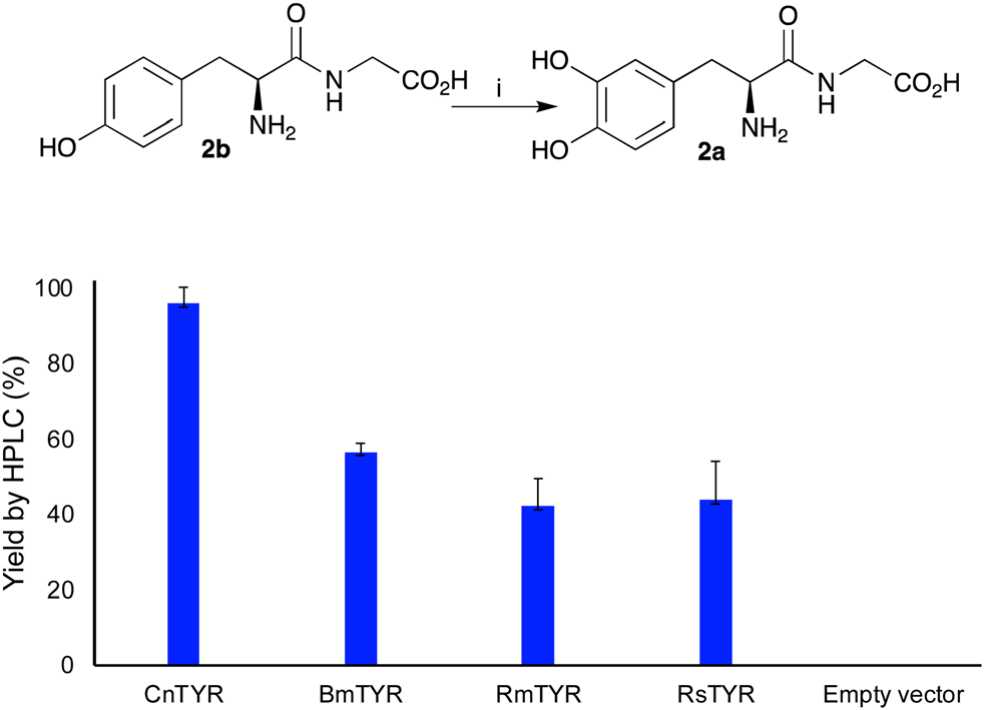
Use of four TYRs to convert 2b into 2a. Reaction conditions: (i) TYRs lysates (10%, v/v), 2b (1 equiv.), sodium ascorbate (2 equiv.), in 0.2 M KPi buffer (pH 6), 37 °C, 18 h. A negative control was carried out using cell lysates containing an empty pET-29 vector.

To convert peptides with an N-terminal tyrosine residue into DOPA-peptides, the use of TYR enzymes was then explored for use in subsequent PSRs. Previous work, has reported the hydroxylation of tyrosine residues using mushroom TYR for applications in alkylation reactions.^10^ Here, four recombinant TYRs, overexpressed in *E. coli*, with good monophenolase activity were used, *Candidatus* nitrosopumilus tyrosinase (*Cn*TYR), *Ralstonia solanacearum* tyrosinase (*Rs*TYR), *Bacillus megaterium* tyrosinase (*Bm*TYR) and *Rhizobium meliloti* tyrosinase (*Rm*TYR).^11^ When TYR enzyme lysates (10% v/v) were used with Tyr-Gly 2b, 2a was formed in 96% yield by HPLC analysis (against product standards) for *Cn*TYR (Fig. 1). Indeed, preliminary docking experiments with *Cn*TYR and 2b (Fig. S1, ESI†) highlighted that it readily fitted into the active site. The other three, *Rs*TYR, *Bm*TYR and *Rm*TYR gave lower yields (40–60%), so *Cn*TYR was explored further.

**Table 1:** Chemoenzymatic one-pot reactions with 2b and aromatic aldehydes 1a–1h^a^

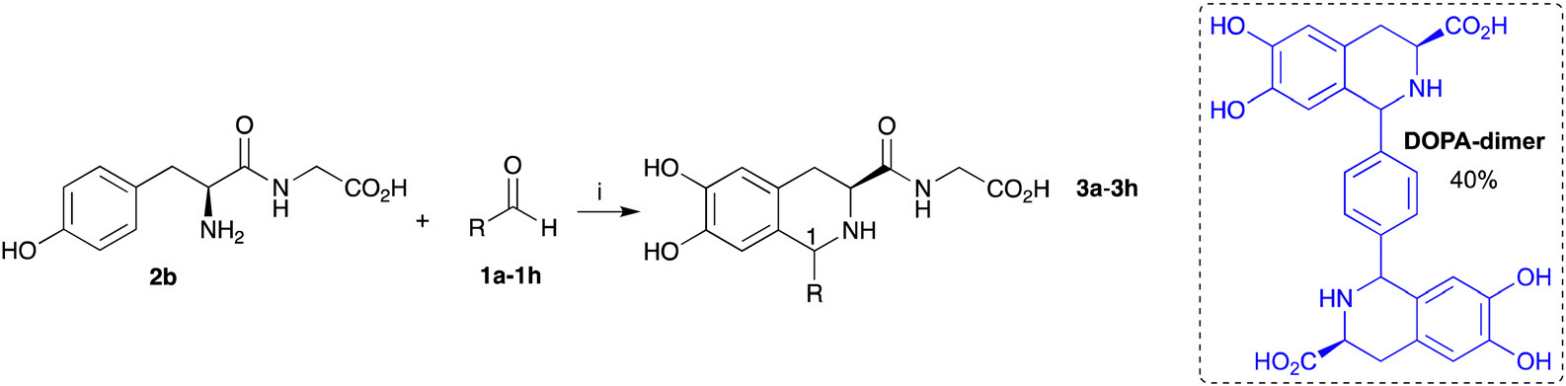		
Aldehyde	R	3 Yield^b^ (ratio 1*R*,3*S* : 1*S*,3*S*)
1a	CH_2_Ph	3a 75% (1 : 1)
1b	Ph	3b 43% (3 : 1)
1c	4-ClC_6_H_4_	3c 47% (3 : 1)
1d	4-BrC_6_H_4_	3d 62% (3 : 1)
1e	2-BrC_6_H_4_	3e 52% (1 : 1)
1f	4-HOC_6_H_4_	3f 0%
1g	4-MeOC_6_H_4_	3g 10% (4 : 1)
1h	4-CHO-C_6_H_4_	3h 30% (2 : 1) + 3h-dimer by MS
1i	1-Pyrene	3i 50% (1 : 1)
1j	(CH_2_)_3_-1-pyrene	3j 60% (1 : 1)

a Reaction conditions: (i) 2b and aldehydes 1a–1g (1 : 1.5) (1h a ratio of 3 : 1), sodium ascorbate (3 equiv.), *Cn*TYR lysates (10%, v/v) in 0.2 M KPi buffer/CH_3_CN (10%, v/v), pH 6.0, 37 °C, 18 h. For 1i and 1j, 2b (1 equiv.) was converted into 2a with *Cn*TYR (10%, v/v), sodium ascorbate (3 equiv.) in KPi buffer (0.2 M, pH 6.0), 37 °C, 18 h, then 2a formed was reacted with 1i or 1j. For 1i (1.5 equiv.), sodium ascorbate (3 equiv.), in KPi buffer/MeOH/CH_3_CN (1 : 1 : 1), at pH 6.0 (adjusted with 0.2 M KH_2_PO_4_ and K_2_HPO_4_), 50 °C, 18 h. With 1j (1.5 equiv.) KPi buffer/MeOH/EtOAc (5 : 3 : 2), at pH 6.0 (adjusted as above).

b Yields were determined by analytical HPLC (against product standards). Diastereoselectivities were determined by HPLC and ^1^H NMR spectroscopy with assignment of the sterochemistry using NOEs (see ESI).^30^

The integration of the biocatalytic and chemical steps in a one-pot reaction has many advantages in terms of improved efficacy.^29^ To build the cascade using *Cn*TYR followed by the PSR, 2b and aldehydes 1a–1h were used to determine whether THIAs 3a–3h could be formed in one-pot reactions (Table 1). Reactions were performed for 18 h, with all components present. To provide a balance between aldehyde solubility and *Cn*TYR activity, 10% acetonitrile was used together with KPi buffer to promote the PSR. Again, enzyme lysates were used for ease of preparation. Dipeptide 2b generated 2a in the reaction, which then reacted with 1a to give 3a in 75% yield (and 1 : 1 ratio of isomers). Interestingly the yield was higher than when using DOPA-Gly 2a directly as the starting material (Scheme 2). This may have been due to the lower reaction temperature and *in situ* production of 2a which then spontaneously cyclised with 1a to give 3a, avoiding side product formation due to the oxidation of 2a.

Aromatic aldehydes 1b–1h were then used in this one-pot cascade. Benzaldehyde 1b and halogenated aldehydes 1c–1e gave the corresponding THIAs 3b–3e in good yields (43–62%) over two steps. In contrast, 4-hydroxyaldehyde 1f contains an electron donating group, making the carbonyl less electrophilic and also making the substrate susceptible to oxidation by *Cn*TYR; thus no THIA products were formed (Table 1). With 4-methoxybenzaldehyde 1g, 3g was formed in 10% yield. Interestingly, reactions with 1b–1d and 1g showed some preference for the (1*R*,3*S*)-configured products (3 : 1 or 4 : 1). However, the reaction with 1e resulted in a lower stereoselectivity, which may be due to unfavourable steric interactions.^30^ Initially for dialdehyde 1h, the ratio of 2b : 1h used was 3 : 1 as potentially a dimer could be formed, however this gave the monomer 3h in 30% yield as a 2 : 1 mixture of diastereoisomers. A dimeric product 3h-dimer was detected by high resolution mass spectrometry (HRMS) but could not be isolated due to the small amounts formed. The reaction was explored further and when using l-DOPA and 1h (ratio 2 : 1) the corresponding DOPA-dimer was formed exclusively in 40% yield by HPLC (Table 1, for further details see the ESI†). It is possible that the amide at C-3 in 3h makes the intermediate more sterically crowded, stopping the second PSR from occurring to give the dimer.

To further demonstrate the chemoenzymatic cascade, fluorescent aldehydes were then used to selectively incorporate fluorophores at the N-terminus of the model dipeptide. 1-Pyrenecarboxaldehyde 1i was used as purchased, and 4-(pyren-1-yl)butanal 1j was synthesised from the corresponding acid.^31^ In this cascade, due to the poor aqueous solubility of the aldehydes, 2b was firstly converted into 2a, which was then used in the second step with the aldehydes (in KPi buffer/MeOH/CH_3_CN (1 : 1 : 1)) at 50 °C for 18 h. The corresponding products 3i and 3j were synthesised in 50% and 38% yields, respectively. Notably, the reaction with 1j in KPi buffer/MeOH/EtOAc (5 : 3 : 2) gave 3j in much higher yield (60%, Table 1) compared to that in KPi buffer/MeOH/CH_3_CN (1 : 1 : 1), reflecting the poor solubility of hydrophobic 1j in aqueous media, and the importance of solvent selection for the PSR reactions. Both 3i and 3j were formed as mixed diastereomers at C-1 in ratios of 1 : 1.

To demonstrate the application of the one-pot chemoenzymatic cascade with a pentapeptide, Leu-enkephalin 2c was synthesised as previously reported.^32^ Preliminary docking experiments also confirmed a productive conformation with *Cn*TYR (Fig. S2, ESI†). Pentapeptide Tyr-Gly-Gly-Phe-Leu 2c, with an N-terminal Tyr-residue, is an endogenous opioid neurotransmitter found naturally in the brains of animals, including humans.^33^ The reaction conditions developed with 2b were initially used with 2c and aldehydes 1a–1e to give the corresponding THIA-peptides 4a–4e (Table 2) in 25–77% yields (by analytical HPLC against standards). The reaction was carried out on a larger scale and the products purified for characterisation purposes and to determine diastereoselectivities at C-1.

**Table 2:** Chemoenzymatic one-pot reactions with 2c and aromatic aldehydes 1a–1e^a^

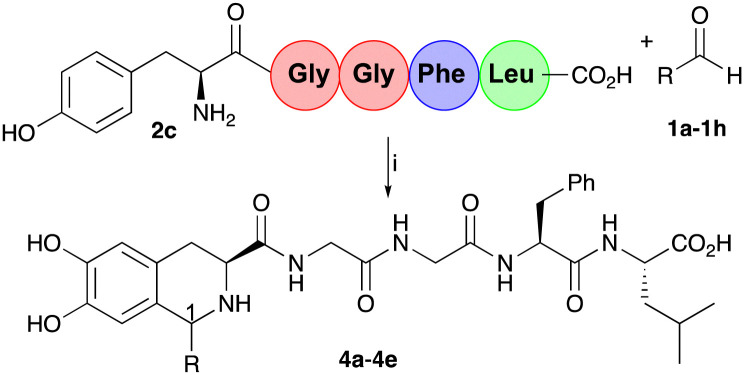		
Aldehyde	R	4 Yield^b^ (ratio 1*R*,3*S* : 1*S*,3*S*)
1a	CH_2_Ph	4a 40% (1 : 1)
1b	Ph	4b 50% (3 : 1)
1c	4-ClC_6_H_4_	4c 25% (3 : 1)
1d	4-BrC_6_H_4_	4d 36% (3 : 1)
1e	2-BrC_6_H_4_	4e 77% (1 : 1)

a Reaction conditions: (i) 2b/2c and aldehydes (1 : 1.5) (other than 1 h with a ratio of 3 : 1), sodium ascorbate (3 equiv.), *Cn*TYR lysates (10%, v/v) in 0.2 M KPi buffer/CH_3_CN (10%, v/v), pH 6.0, 37 °C, 18 h.

b Yields were determined by analytical HPLC (against product standards). Diastereoselectivities were determined by HPLC and ^1^H NMR spectroscopy with assignment of the sterochemistry using NOEs (see ESI).^30^

Product 4a was formed as a mixture of isomers (1 : 1) at C-1, comparable to the selectivity in 3a. Interestingly, 4b–4d were again formed in a 3 : 1 ratio for (1*R*,3*S*):(1*S*,3*S*), which was similar to the reaction selectivities when using 2b.

**Fig. 2:**
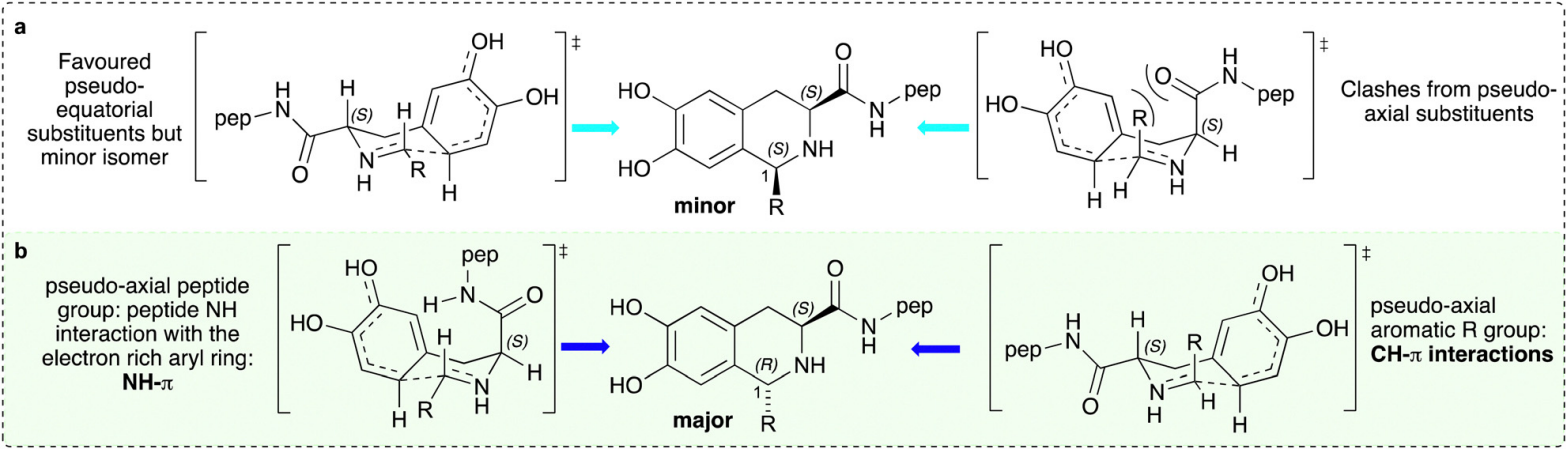
Consideration of intermediates to give THIAs 3b–3d and 4b–4d in ratios of 3 : 1, (1*R*,3*S*):(1*S*,3*S*). (a). Possible transition states leading to the major isomer. (b). Possible transition states leading to the minor isomer. ‘Pep’ is the peptide.

The stereochemical outcome is interesting and with a view to providing a preliminary rationalisation for these observations, potential intermediates leading to the major and minor products were considered for both 3b–3d and 4b–4e (Fig. 2). If the major product formed was due to steric considerations only, then the (1*S*,3*S*)-isomer would be formed preferentially with the peptide side chain and R-aryl group adopting pseudo-equatorial conformations (Fig. 2a). However, this is not the case so is likely that non-covalent π-interactions are important.^34^ These could either be NH–π interactions between the peptide NH and catechol ring, or CH–π interactions between the R-aryl ring CH moiety and catechol ring, where both the peptide chain and R-aryl group adopt a pseudo-axial orientation (Fig. 2b).^34,35^ On the basis of the improved selectivity with aryl *versus* phenylacetaldehyde R groups, it could be possible that CH–π interactions may predominate.

### Peptide–peptide couplings

With successful coupling of di- and pentapeptides with aromatic species, peptide–peptide couplings were then explored using 2b together with di- and tri-peptide aldehydes. While PSRs have been incorporated into chemical peptide ligation strategies using N-terminal tryptophan-peptides and aldehyde-tagged peptides, N-terminal tyrosine residues have not been used. Applying this new chemoenzymatic approach for peptide coupling, *N*-Boc-Valinyl alanal 5a, *N*-Boc-phenylalanyl alanal 5b, and *N*-Boc-phenylalanylvalinyl alanal 5c were first prepared *via* reduction of the corresponding Weinreb amides.^36,37^ These were used as Boc-protected aldehydes in initial experiments in order to avoid intermolecular aldehyde imine formation.

**Scheme 3:**
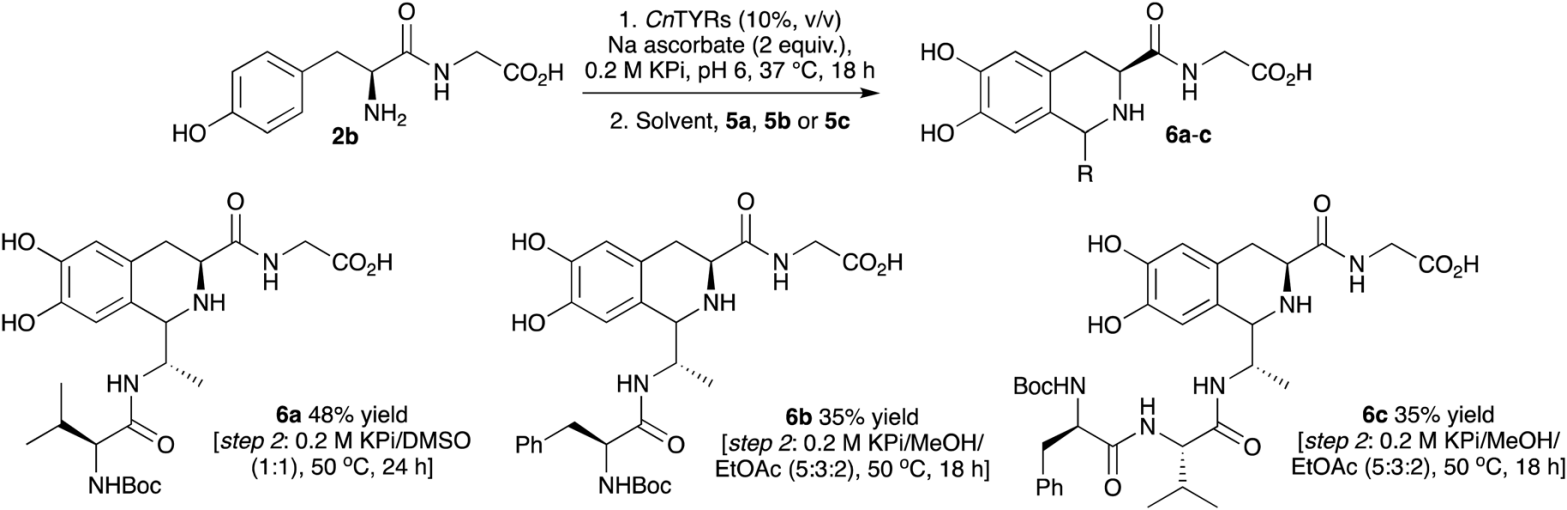
Chemoenzymatic one-pot two step reaction with 2b and 5a–c (1 : 1.5) to products 7a–c. Yields were determined by analytical HPLC (against product standards). Further details are in the ESI.†

The reactions with 2b and peptide aldehydes 5a–5c were initially explored as a one-pot procedure, however little product was observed. Therefore, a one-pot, two-step procedure was developed. First, the conversion of 2b into 2a using *Cn*Tyr as before was carried out. Then, 5a was added to generate a solvent composition of 0.2 M KPi buffer, 10% CH_3_CN and the reaction was left for a further 24 h. This gave the coupled peptide 6a in 30% yield (Scheme 3). To optimise this sequence, other solvent mixtures (*via* solvent addition) and reaction temperatures were also employed for the second step. The best conditions were found to be a solvent mixture of 0.2 M KPi buffer/DMSO (1 : 1) (to enhance the solubility of the aldehyde) and performing the reaction at 50 °C over 24 h to give 6a in 48% yield. For 5b and 5c, a mixture of 0.2 M KPi buffer/MeOH/EtOAc (5 : 3 : 2), was found to be effective in the second step and 6b and 6c were formed in 35% yield (Scheme 3). In all cases a 1 : 1 mixture of isomers at C-1 was generated.

Since the reactive residue is an N-terminal tyrosine, this approach provides a site-selectively conjugation method for peptide ligation and the addition of functional motifs. The cascade strategy here is complementary to existing peptide-ligation methods but uses a new strategy, the hydroxylation of tyrosine residues using *Cn*TYR with subsequent coupling to aldehydes under mild conditions.

## Conclusions

In summary, a new approach has been developed for coupling N-terminal tyrosine-containing peptides with aldehydes, utilising a tyrosinase enzyme to convert l-Tyr to l-DOPA residues, with a subsequent Pictet–Spengler reaction. This new chemoenzymatic coupling strategy was demonstrated using di- and pentapeptides with aromatic aldehydes, fluorescent aldehydes and peptide aldehydes. In addition, in several cases stereoselectivities of up to ∼3 : 1 were observed in the PSR coupling reaction. However, it was noted that poorly water soluble substrates could limit some applications. Further studies are also required to better understand the stereoselectivities observed. Despite this, both one-pot and one-pot, two-step reaction cascades were developed under mild reaction conditions. This approach has many applications as a peptide-ligation strategy under physiological conditions.

## Author contributions

Y.N. investigated the chemoenzymatic cascades and Y.N. and Y.W. developed the enzymatic methodologies. The project conceptualisation was by all authors and supervised by A.B.T, J.M.W. and H.C.H. The manuscript original-draft was written by Y.N. and H.C.H. The manuscript has been reviewed and edited by all contributing authors.

## Conflicts of interest

There are no conflicts to declare.

## Supplementary Material

CB-004-D2CB00237J-s001
