# Supplementary material for: The use of tyrosinases in a chemoenzymatic cascade as a peptide ligation strategy
Source: RSC Chem Biol. 2022 Dec 12;4(2):132–7. doi: 10.1039/d2cb00237j (PMC9906322; doi:10.1039/d2cb00237j)
Supplement: CB-004-D2CB00237J-s001 [file CB-004-D2CB00237J-s001.pdf]

Electronic Supplementary Material (ESI) for RSC Chemical Biology.
This journal is © The Royal Society of Chemistry 2022

Supporting Information

The use of tyrosinases in a chemoenzymatic cascade as a peptide ligation strategy

Yeke Ni, Yu Wang, Alethea B. Tabor, John M. Ward and Helen C. Hailes*

Table of Contents

1. ***General Methods***......3

2. ***Protein Expression and Cell Lysates Preparation***......4

3. ***HPLC Methods***......6

4. ***General Synthetic Procedures***......7

5. ***Procedures and Characterization***......8

6. ***Molecular Docking Figures***......27

7. ***Abbreviation***......29

8. ***References***......30

1. General Methods

Chemicals: All chemicals were purchased from commercial suppliers (Sigma-Aldrich, Acros Organics, Alfa Aesar etc.) and used as received. All chemicals were purchased in the highest purity available. Thin layer chromatography (TLC) was carried out with Merck TLC silica gel 60 F254 pre-coated plates, or Geduran® Si 60 silica (43-60 μm) and products were visualised using UV light (254, 360 nm), potassium permanganate, phosphomolybdic acid, or ninhydrin staining solutions.

Nuclear Magnetic Resonance (NMR) spectroscopy: ^{1}H and ^{13}C NMR spectra were recorded using Bruker Advance 500, 600 and 700 MHz spectrometers at 298 K. The chemical shifts (in ppm) were determined relative to tetramethylsilane (TMS) set at 0 ppm and referenced to residual, protonated NMR solvents. Coupling constants in ^{1}H NMR spectra were defined as *J* and measured in Hertz (Hz), and are described as singlet (s), doublet (d), triplet (t), doublet of doublets (dd) etc.

Infrared spectra: Infrared spectra were recorded on a Perkin Elmer Spectrum 100 FTIR spectrometer.

Liquid chromatography–mass spectrometry (LC-MS): LC-MS was performed on an Agilent 1100 Series System with a Finnigan LTQ mass spectrometer. An ACE 5 C18 reverse phase column (50 mm × 2.1 mm, 5 μm) was adopted with a mobile phase of eluent A (H_2O with 0.1% (v/v) formic acid) and eluent B (acetonitrile) over 5 min with a flow rate of 0.2 mL/min, following a gradient of A/B = 95%/5% to 5%/95% at 0-4 min, A/B = 5%/95% to 95%/5% at 4-4.5 min, A/B = 95%/5% at 4.5-5 min. The sample injection volume was 10 μL. Chemical compounds were measured in a positive ion mode, and the operating conditions of the ESI interface were set to a capillary temperature 300 °C, capillary voltage 9 V, spray voltage 4 kV, sheath gas 40, auxillary gas 10, sweep gas 0 arbitrary units.

High-resolution mass spectrometry (HRMS): High-Resolution Mass spectrometry experiments were performed by the UCL Chemistry Mass spectrometry service, using EI or CI on a MAT900 or a Waters LCT Premier Q-TOF for ESI.

2. Protein Expression and Cell Lysates Preparation

Tyrosinase expression: A *Cn*TYR glycerol stock (*E. coli* BL21 (DE3) was transferred to 5 mL of LB media supplemented with 50 µg/mL kanamycin and grown at 37 °C, 250 rpm overnight. The culture was transferred to a 500 mL baffled shaking flask containing 100 mL TB media supplemented with 50 µg/mL kanamycin and shaken at 37 °C, 250 rpm until OD_{600} = 0.9. Enzyme expression was initiated by the addition of 1 mM IPTG (Sigma-Aldrich) and 1 mM $CuSO_4$ (Sigma-Aldrich) to the culture. The mixture was shaken at 25 °C, 250 rpm for 24 h, and centrifuged at 5000 rpm for 30 min. The cells were stored at -20 °C.

Cell lysates of *Cn*TYR: Cell pellets (from 50 mL cultures) were resuspended in 5 mL buffer (KPi, pH 6.0) and lysed by 10 cycles of sonication on ice (10 s on, 10 s off, 12 watts output). The resulting mixture was centrifuged at 4 °C, 5000 rpm for 30 min. The supernatant was stored at -20 °C. The concentration of supernatant protein was measured following the standard Bradford procedure. The samples were duplicated and the average OD_{595} were used for cell lysate concentration calculations.

Cell lysates of other TYR enzymes (*Rs*TYR, *Bm*TYR and *Rm*TYR) were generated using previously reported procedures.[1]

Tyrosinase sequence

Tyrosinase from *Candidatus Nitrosopumilus salaria* BD31(*Cn*TYR, Gnene Bank: GenBank: EIJ65432.1)

```
MVRKNASSLNPIERENFCKAVLTLKNTKIPGHALNRYDEFVAIHFGVTSRERANLPIGDGAHGNSGFLPWHREFL
CRFEHALKSVDPTVSLPYWDWSSGDTSDTIDIFNDDFMGPAGTVNSGYFSGTGNSFNSNRPWIVHPSLDQTSPGQ
PPLGSTLIRNSNLLSASTLNYLMDLGEMARDSLNESTYNAFRSTLEHPPHNHVHGVTVQGHMGWMTSPNDPIFFL
HHANVDRLWAEWQRTHPGSSNYTPNATEPYGVHLNDPMWPWQGADTTVTTRTHTDSNASLNTLLPSFSTADLVTP
NDVLDHIQRCGPYDTDPISKPKEFEKIPKEIIKEIIKDKEKEFGDKNPKEIIKEIIKDKEKEFGDKNPKEIIKEI
IKDKEKEFGDKNPKEIKEIIKDKEKEFGDKNPKEIKEIIKDKEKEFGDKNPKEIIETGDIKIENNKDVVEILSTP
STTVSSPKHPKEQSKETLEITNTLFDPLSKINHRLDMLENEIKGTAFIKSTERPNITKRAISKNTSTKKTTRKKT
KNTKNTMPKKSNTSKRKRISHHHHHH
```

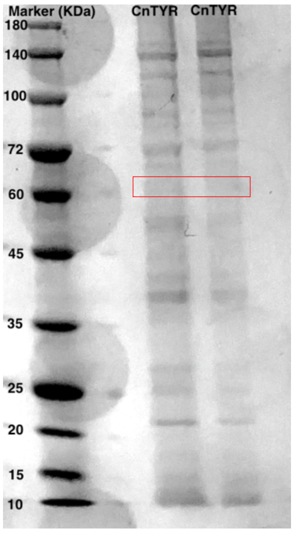

Figure 1. SDS gel for *Cn*TYR lysates. From left to right: protein marker (GeneTex), 5 μL lysates, 2 μL lysates. *Cn*TYR: 61.2 KDa.

3. HPLC Methods

Analytical high-performance liquid chromatography (HPLC): Reaction analysis was carried out on an Agilent 1260 Infinity HPLC system comprising of a G1329B autosampler, a G1311C quaternary pump, a 1260 G1316A column oven and a 1260 G1314F variable wavelength detector, equipped with an ACE 5 C18 reverse phase column (150 mm × 4.6 mm) with acetonitrile and water (0.1% TFA, v/v) as mobile phases. The flow rate was 1 mL/min at 30 °C, and the injection volume was 10 µL. The UV absorbance was measured at 280 nm.

Method 1: mobile phase (A: water with 0.1% TFA; B: acetonitrile) in a gradient of A/B = 90%/10% at 0-1 min, A/B = 90%/10% to A/B = 30%/70% at 1-6 min, 100% B at 6-6.5 min, and A/B = 90%/10% at 6.5-10 min.

Method 2: mobile phase (A: water with 0.1% TFA; B: acetonitrile) in a gradient of A/B = 90%/10% at 0-1 min, A/B = 90%/10% to A/B = 30%/70% at 1-20 min, A/B = 30%/70% to A/B = 5%/95% at 20-20.2 min, 95% B at 20.2-23 min, A/B = 5%/95% to A/B = 90%/10% at 23-23.1 min, and A/B = 90%/10% at 23.1-25 min.

Method 3: mobile phase (A: water with 0.1% TFA; B: acetonitrile) in a gradient of A/B = 95%/5% at 0-1 min, A/B = 95%/5% to A/B = 60%/40% at 1-35 min, A/B = 60%/40% to A/B = 30%/70% at 35-35.1 min, 70% B at 35.1-38.5 min, A/B = 30%/70% to A/B = 95%/5% at 38.5-39 min, and A/B = 95%/5% at 39-40 min.

Preparative HPLC: The HPLC purification was performed on Agilent 1200 Infinity series HPLC system comprising of a G1361A prep pump, a G2260A autosampler, a G1364A fraction collector and a G7165 multiple wavelength detector. A Vydac 218TP1022 C18 column (10 µm, 2.2 cm ID x 25 cm L) was used with acetonitrile (0.1% TFA, v/v) and water (0.1% TFA, v/v) as mobile phases and a flow speed at 8 mL/min at 25 °C. The UV absorbance was measured at 214 and 280 nm.

Method 4: mobile phase (A: water with 0.1% TFA; B: acetonitrile) in a gradient of A/B = 95%/5% at 0-3 min, A/B = 95%/5% to A/B = 5%/95% at 3-20 min, A/B = 5%/95% at 20-22 min, A/B = 5%/95% to A/B = 95%/5% at 22-23 min, and A/B = 95%/5% at 23-28 min.

Method 5: mobile phase (A: water with 0.1% TFA; B: acetonitrile) in a gradient of A/B = 95%/5% at 0-3 min, A/B = 95%/5% to A/B = 60%/40% at 3-42 min, A/B = 60%/40% to A/B 5%/95% at 42-42.5 min, A/B = 5%/95% at 42.5-46.5 min, A/B = 5%/95% to A/B = 95%/5% at 46.5-47 min, and A/B = 95%/5% at 47-50 min.

4. General Synthetic Procedures

Procedure A. Pictet-Spengler chemoenzymatic reaction

The N-terminal tyrosine peptide (1 equiv.), aldehyde (1.5 equiv.) and sodium ascorbate (3 equiv.) were mixed in 10 mL of a 1:9 mixture of acetonitrile/potassium phosphate buffer (0.2 M, pH 6.0). *Cn*TYR enzyme (10%, *v/v*) was added. The resulting solution was stirred at 37 °C for 18 h. Afterwards, the reaction was concentrated *in vacuo* and resuspended in 2 mL of methanol. The resuspension was filtered and purified by a preparative HPLC. Fractions were collected, concentrated, and freeze-dried to give target products.

Procedure B. Pictet-Spengler chemoenzymatic reactions with terephthalaldehyde

The N-terminal tyrosine peptide (3 equiv.), aldehyde (1 equiv.) and sodium ascorbate (6 equiv.) were mixed in 10 mL of a 1:9 mixture of acetonitrile/potassium phosphate buffer (0.2 M, pH 6.0). *Cn*TYR enzyme (10%, *v/v*) was added. The resulting solution was stirred at 37 °C for 18 h. Afterwards, the reaction was concentrated *in vacuo* and resuspended in 2 mL of methanol. The resuspension was filtered and purified by a preparative HPLC. Fractions were collected, concentrated, and freeze-dried to give the target products.

Procedure C. General peptide coupling reaction

Acid (1 equiv.), amine (1.1 equiv.) and HBTU (1.2 equiv.) were mixed in DMF (10 mL) and cooled to 0 °C. DIPEA (5 equiv.) was added. The reaction was stirred under argon at 0 °C for 30 min, then at room temperature for 18 h. The reaction was concentrated *in vacuo* and redissolved in EtOAc (10 mL). The organic layer was then washed with 1 M HCl (3 × 10 mL), saturated $NaHCO_3$ (3 × 10 mL) and brine (10 mL), and then dried over anhydrous Na_2SO_4, and concentrated *in vacuo* to give the product.

Procedure D. DOPA-Gly formation from Tyr-Gly with TYRs

Tyr-Gly **2b** (1 equiv.) and sodium ascorbate (2-3 equiv.) were mixed in 0.2 M KPi buffer (pH 6.0), and 10% v/v *Cn*TYR lysates were added. The reaction was shaken in an incubator at 37 °C for 18 h. The reaction was quenched by adding 0.1% TFA. Precipitates were removed by centrifugation. The supernatant was concentrated *in vacuo* to give **2a** as a yellow oil.

5. Procedures and Characterization

(2*S*)-(2-Amino-3-(3',4'-dihydroxyphenyl)propanoyl)glycine (L-DOPA-Gly) 2a[2,3]

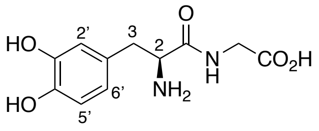

Procedure 1: To a solution of L-DOPA (500 mg, 2.54 mmol) in dioxane/water (1:1, 10 mL), NEt_3 (0.530 mL, 3.81 mmol) and di-*tert*-butyl-dicarbonate (600 mg, 2.74 mmol) were added. The mixture was stirred at 0 °C for 30 min, then at room temperature overnight. The resulting solution was concentrated and re-dissolved in H_2O (5 mL), washed with EtOAc (3 x 10 mL). The aqueous layer was acidified to pH 1 by 1 M HCl and washed with EtOAc (3 x 10 mL). The combined organic layers were dried over anhydrous Na_2SO_4 and concentrated *in vacuo* to yield Boc-DOPA (0.453 g, 60% yield) as a brown oil. To a solution of Boc-DOPA (762 mg, 2.56 mmol) in CH_2Cl_2 (14 mL), N-Gly-O^{t}Bu hydrochloride (320 mg, 2.56 mmol), EDC (490 mg, 2.56 mmol) and HOBT (340 mg, 2.56 mmol) were added. The mixture was cooled to 0 °C, and DIPEA (0.440 mL, 2.56 mmol) was added. The reaction solution was stirred at 0 °C for 30 min, then at room temperature overnight. The resulting solution was washed with H_2O (3 x 10 mL), 2 N HCl (3 x 10 mL), saturated $NaHCO_3$ (3 x 10 mL), and dried over anhydrous Na_2SO_4. The organic layer was concentrated *in vacuo* to obtain Boc-DOPA-Gly-O^{t}Bu (0.289 g, 26% yield) as a yellow oil. Protected dipeptide (1.18 g, 2.90 mmol) was then dissolved in CH_2Cl_2 (6 mL), and the solution was cooled to 0 °C. TFA (6.0 mL, 7.8 mmol) was added. The reaction was stirred at room temperature for 5 h. The resulting solution was concentrated *in vacuo* to remove excess TFA and solvent to give N-DOPA-Gly-OH **2a** (0.61 g, 83% yield) as a yellow oil. $[\alpha]_D^{26}$ +20.0 (c 1.0, CH_3OH); ν_{max} (solution in CH_3OH)/cm^{-1} 3335, 3296, 1633; ^{1}H NMR (700 MHz; D_2O) δ 6.82 (1H, d, *J* = 8.1 Hz, 5'-H), 6.74 (1H, d, *J* = 2.2 Hz, 2'-H), 6.66 (1H, dd, *J* = 8.1, 2.2 Hz, 6'-H), 4.17 (1H, t, *J* = 7.2 Hz, 2-H), 3.97 (1H, d, *J* = 18.0 Hz, NHC*H*H), 3.89 (1H, d, *J* = 18.0 Hz, NHCH*H*), 3.06 (1H, dd, *J* = 14.2, 7.2 Hz, 3-*H*H), 3.00 (1H, dd, *J* = 14.2, 7.2 Hz, 3-H*H*); ^{13}C NMR (176 MHz; D_2O) δ 173.3, 170.2, 144.8, 144.1, 126.7, 122.4, 117.6, 117.0, 55.0, 41.5, 36.6. *m/z* [ES+] 255 ([M+H]$^+$, 100%); *m/z* [HRMS ES-] found [M-H]$^-$ 253.0824. [$C_{11}H_{14}N_2O_5$-H]$^-$ requires 253.0829.

Procedure 2: **2b** (100 mg, 0.420 mmol) and sodium ascorbate (168 mg, 0.840 mmol) were mixed in 0.2 M KPi buffer (pH 6.0, 10 mL), 10% v/v *Cn*TYR lysates were added. The reaction was shaken in the incubator at 37 °C, 18 h. After reaction, 0.1% TFA was added and centrifuged. The supernatant was concentrated *in vacuo* to give **2a** (105 mg, 99%) as a yellow oil. The characterisation data was identical to that above.

L-Tyrosyl glycine (L-Tyr-Gly) 2b[2,4]

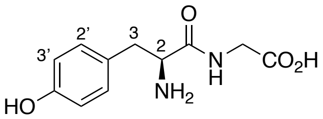

The protected dipeptide was prepared according to **Procedure C** using Boc-Tyr(^{t}Bu)-OH (0.17 g, 0.50 mmol) and *tert*-butyl glycine hydrochloride (92 mg, 0.55 mmol). The product was purified using silica gel column chromatography (petroleum ether 40-60/ethyl acetate, 4:1 to 1:1) to give Boc-Tyr(^{t}Bu)-Gly-O^{t}Bu (0.19 g, 86%) as a yellow oil. Rf = 0.5 (petroleum ether 40-60/ethyl acetate, 2:1). Boc-L-Tyr(^{t}Bu)-Gly-O^{t}Bu (195 mg, 0.430 mmol) was then dissolved in CH_2Cl_2 (10 mL), cooled to 0 °C, and TFA (10 mL) was added. The reaction was stirred under argon at 0 °C for 30 min, then at room temperature for 4 h. The reaction was then concentrated *in vacuo* to give the crude product. The product was further purified by preparative HPLC (**Method 4**, Rt = 13.2 min) to give **2b** (150 mg, 90% yield) as a colourless oil. $[\alpha]_D^{26}$ +31.7 (c 0.6, CH_3OH); ν_{max} (solution in CH_3OH)/cm^{-1} 3326, 3288, 1643, 1014; ^{1}H NMR (700 MHz; CD_3OD) *δ* 7.12 (2H, d, *J* = 8.5 Hz, 3'-H), 6.77 (2H, d, *J* = 8.5 Hz, 2'-H), 4.04 (1H, dd, *J* = 8.2, 6.0 Hz, 2-H), 3.98 (1H, d, *J* = 17.8 Hz, NHC*H*H), 3.90 (1H, d, *J* = 17.8 Hz, NHCH*H*), 3.16 (1H, dd, *J* = 14.3, 6.0 Hz, 3-*H*H), 3.07 (1H, dd, *J* = 14.3, 8.2 Hz, 3-H*H*); ^{13}C NMR (151 MHz; CD_3OD) *δ* 172.7, 170.2, 158.4, 125.9, 121.6, 116.8, 56.0, 41.6, 37.6; *m/z* [ES+] 239 ([M+H]$^+$, 100%); *m/z* [HRMS ES+] found [M+H]$^+$ 239.1026. $[C_{11}H_{14}N_2O_4+H]^+$ requires 239.1026.

((3*S*)-1-Benzyl-6,7-dihydroxy-1,2,3,4-tetrahydroisoquinoline-3-carbonyl)glycine 3a

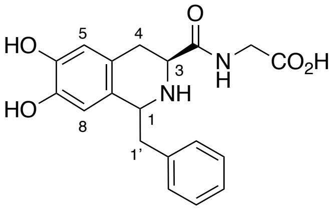

Compound **3a** (both isomers) were prepared according to **procedure A** using **2b** (23.8 mg, 0.100 mmol) and phenylacetaldehyde **1a** (17.5 µL, 0.150 mmol). The product was purified by preparative HPLC (**Method 5,** Rt = 27.4 min) to give **3a** (the two diastereomers were assigned from the ^{1}H NMR NOE data) as a white solid (9.7 mg, 27% yield) (75% HPLC yield against product standards). M.p. 250-260 °C; ν_{max} (neat)/cm^{-1} 3033, 1664, 1186, 1130; (1*R*,3*S*) (53% ratio): ^{1}H NMR (600 MHz; CD_3OD) *δ* 7.42-7.17 (5H, m, Ph-H), 6.63 (1H, s, 5-H), 6.04 (1H, s, 8-H), 4.66 (1H, dd, *J* = 8.8, 4.9 Hz, 1-H), 4.32 (1H, dd, *J* = 11.6, 5.7 Hz, 3-H), 4.04 (1H, d, *J* = 17.8 Hz, NHC*H*H), 4.00 (1H, d, *J* = 17.8 Hz, NHCH*H*), 3.34 (1H, dd, *J* = 13.3, 4.9 Hz, 1'-*H*H), 3.26 (1H, dd, *J* = 16.9, 5.7 Hz, 4-*H*H), 3.15 (1H, dd, *J* = 13.3, 8.8 Hz, 1'-H*H*), 3.04 (1H, dd, *J* = 16.9, 11.6 Hz, 4-H*H*); ^{13}C NMR (151 MHz; CD_3OD) *δ* 172.6, 170.2, 147.2, 145.5, 130.6, 130.3, 130.0, 128.7, 122.7, 121.8, 115.9, 114.9, 57.2, 52.3, 41.7, 41.5, 30.1. (1*S*,3*S*) (47%

ratio): ^{1}H NMR (600 MHz; CD_3OD) δ 7.42-7.17 (5H, m, Ph-H), 6.85 (1H, s, 5-H), 6.66 (1H, s, 8-H), 4.70 (1H, dd, J = 9.7, 4.9 Hz, 1-H), 4.03-4.00 (1H, m, 3-H), 4.00 (1H, d, J = 18.0 Hz, NHC*H*H), 3.94 (1H, d, J = 18.0 Hz, NHCH*H*), 3.63 (1H, dd, J = 14.5, 4.9 Hz, 1'-*H*H), 3.20-3.10 (2H, m, 4-H_2), 3.04 (1H, dd, J = 14.5, 9.7 Hz, 1'-H*H*); ^{13}C NMR (151 MHz; CD_3OD) δ 172.6, 170.2, 147.2, 146.4, 130.6, 130.3, 130.0, 128.7, 123.5, 123.0, 116.0, 113.5, 57.5, 57.2, 41.6, 40.9, 30.8. *m/z* [ES+] 357 ([M+H]$^+$, 100%); *m/z* [HRMS ES+] found [M+H]$^+$ 357.1445. [$C_{19}H_{20}N_2O_5$+H]$^+$ requires 357.1445.

((3*S*)-6,7-Dihydroxy-1-phenyl-1,2,3,4-tetrahydroisoquinoline-3-carbonyl)glycine 3b

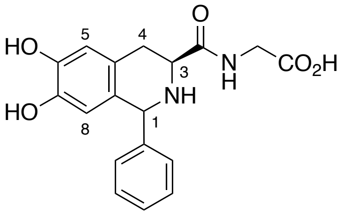

Compound 3b (both isomers) were prepared according to **procedure A** using L-Tyr-Gly (23.8 mg, 0.100 mmol) and benzaldehyde (15.3 µL, 0.150 mmol). The product was purified by preparative HPLC (**Method 5,** Rt = 23.2 min) to give **3b** (the two diastereomers were assigned from the ^{1}H NMR NOE data) as a white solid (10 mg, 29% yield) (43% HPLC yield against product standards). M.p. 160-164 °C; ν_{max} (neat)/cm^{-1} 3079, 1666, 1191; (1*R*,3*S*) (74% ratio): ^{1}H NMR (600 MHz; CD_3OD) δ 7.48-7.30 (5H, m, Ph-H), 6.72 (1H, s, 5-H), 6.35 (1H, s, 8-H), 5.72 (1H, s, 1-H), 4.19 (1H, dd, J = 11.7, 5.2 Hz, 3-H), 3.96 (d, J = 17.8 Hz, 1H, NHC*H*H), 3.92 (1H, d, J = 17.8 Hz, NHCH*H*), 3.28 (1H, dd, J = 16.9, 5.2 Hz, 4-*H*H), 3.15 (1H, dd, J = 16.9, 11.7 Hz, 4-H*H*); ^{13}C NMR (151 MHz; CD_3OD) δ 172.9, 169.3, 147.5, 146.4, 137.8, 131.0, 130.6, 130.2, 123.1, 121.8, 115.5, 115.1, 59.1, 51.2, 41.6, 29.8. (1*S*,3*S*) (26% ratio): ^{1}H NMR (600 MHz; CD_3OD) δ 7.48-7.30 (5H, m, Ph-H), 6.70 (1H, s, 5-H), 6.10 (1H, s, 8-H), 5.59 (1H, s, 1-H), 4.42 (1H, dd, J = 9.4, 8.1 Hz, 3-H), 4.03 (1H, d, J = 17.8 Hz, 1H, NHC*H*H), 4.01 (1H, d, J = 17.8 Hz, NHCH*H*), 3.35-3.25 (2H, m, 4-H_2); ^{13}C NMR (176 MHz; CD_3OD) δ 172.7, 169.5, 146.7, 146.4, 137.8, 131.4, 130.5, 130.4, 123.7, 123.5, 116.0, 115.0, 62.9, 58.0, 41.6, 30.5. *m/z* [ES+] 343 ([M+H]$^+$, 100%); *m/z* [HRMS ES+] found [M+H]$^+$ 343.1289. [$C_{18}H_{18}N_2O_5$+H]$^+$ requires 343.1288.

((3*S*)-1-(4-Chlorophenyl)-6,7-dihydroxy-1,2,3,4-tetrahydroisoquinoline-3-carbonyl)glycine 3c

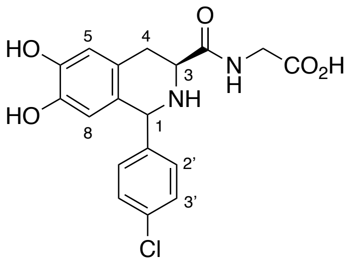

Compound 3c (both isomers) were prepared according to **procedure A** using L-Tyr-Gly (23.8 mg, 0.100 mmol) and 4-chlorobenzaldehyde (21.1 mg, 0.150 mmol). The product was purified by preparative HPLC (**Method 5,** Rt = 31.2 min) to give **3c** (the two diastereomers were assigned from the ^{1}H NMR NOE data) as white solid (6.6 mg, 17% yield) (47% HPLC yield against product standards). M.p. 166 °C; ν_{max} (neat)/cm^{-1} 3064, 1664, 1189, 797; (1*R*,3*S*) (72% ratio): ^{1}H NMR (600 MHz; CD_3OD) δ 7.46 (2H, d, *J* = 8.5 Hz, 3'-H), 7.28 (2H, d, *J* = 8.5 Hz, 2'-H), 6.72 (1H, s, 5-H), 6.35 (1H, s, 8-H), 5.72 (1H, s, 1-H), 4.14 (1H, dd, *J* = 11.7, 5.2 Hz, 3-H), 3.97 (1H, d, *J* = 17.8 Hz, NHC*H*H), 3.90 (1H, d, *J* = 17.8 Hz, NHCH*H*), 3.27 (1H, dd, *J* = 16.9, 5.2 Hz, 4-*H*H), 3.14 (1H, dd, *J* = 16.9, 11.7 Hz, 4-H*H*); ^{13}C NMR (151 MHz; CD_3OD) δ 173.0, 170.1, 147.5, 146.1, 137.1, 136,7, 133.4, 130.2, , 123.1, 121.6, 115.6, 114.9, 58.7, 51.6, 41.9, 30.2. (1*S*,3*S*) (28% ratio): ^{1}H NMR (600 MHz; CD_3OD) δ 7.51 (2H, d, *J* = 8.5 Hz, 3'-H), 7.46 (2H, d, *J* = 8.5 Hz, 2'-H), 6.70 (1H, s, 5-H), 6.08 (1H, s, 8-H), 5.60 (1H, s, 1-H), 4.41 (1H, dd, *J* = 11.1, 6.3 Hz, 3-H), 4.02 (1H, d, *J* = 17.7 Hz, NHC*H*H), 4.01 (1H, d, *J* = 17.7 Hz, NHCH*H*), 3.45-3.35 (2H, m, 4-H_2); ^{13}C NMR (151 MHz; CD_3OD) δ 173.0, 170.1, 146.9, 146.1, 137.1, 136.7, 133.3, 130.2, 123.8, 123.2, 115.5, 114.9, 62.3, 57.8, 42.0, 30.7; *m/z* [ES+] 377 ([M+H]$^+$, 100%); *m/z* [HRMS ES+] found [M+H]$^+$ 377.0900. [$C_{18}H_{17}N_2O_5{}^{35}Cl$+H]$^+$ requires 377.0899.

((3*S*)-1-(4-Bromophenyl)-6,7-dihydroxy-1,2,3,4-tetrahydroisoquinoline-3-carbonyl)glycine 3d

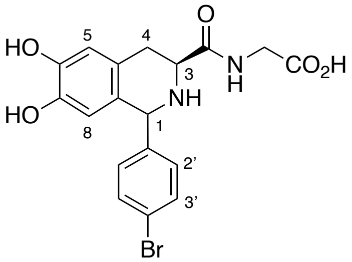

Compound 3d (both isomers) were prepared according to **procedure A** using L-Tyr-Gly (23.8 mg, 0.100 mmol) and 4-bromobenzaldehyde (27.7 mg, 0.150 mmol). The product was purified by preparative HPLC (**Method 5,** Rt = 31.8 min) to give **3d** (the two diastereomers were assigned from the ^{1}H NMR NOE data) as a white solid (7.4 mg, 17% yield) (62% HPLC yield against product standards). M.p. 162 °C; ν_{max} (neat)/cm^{-1} 3066, 1666, 1189, 721; (1*R*,3*S*) (77% ratio): ^{1}H NMR (600 MHz; CD_3OD) δ 7.62 (2H, d, *J* = 8.5 Hz, 3'-H), 7.22 (2H, d, *J* = 8.5 Hz, 2'-

H), 6.72 (1H, s, 5-H), 6.35 (1H, s, 8-H), 5.72 (1H, s, 1-H), 4.14 (1H, dd, *J* = 11.7, 5.2 Hz, 3-H), 3.97 (1H, d, *J* = 17.8 Hz, NHC*H*H), 3.90 (1H, d, *J* = 17.8 Hz, NHCH*H*), 3.27 (1H, dd, *J* = 16.9, 5.2 Hz, 4-*H*H), 3.14 (1H, dd, *J* = 16.9, 11.7 Hz, 4-H*H*); ^{13}C NMR (151 MHz; CD_3OD) δ 173.0, 170.1, 147.5, 146.1, 137.1, 133.4, 133.3, 125.1, 123.1, 121.6, 115.6, 114.9, 58.7, 51.6, 41.9, 30.2. (1*S*,3*S*) (23% ratio): ^{1}H NMR (600 MHz; CD_3OD) δ 7.67 (2H, d, *J* = 8.5 Hz, 3'-H), 7.39 (2H, d, *J* = 8.5 Hz, 2'-H), 6.70 (1H, s, 5-H), 6.08 (1H, s, 8-H), 5.60 (1H, s, 1-H), 4.41 (1H, dd, *J* = 11.1, 6.3 Hz, 3-H), 4.03 (1H, d, *J* = 17.7 Hz, NHC*H*H), 3.99 (1H, d, *J* = 17.7 Hz, NHCH*H*), 3.45-3.35 (2H, m, 4-H_2); ^{13}C NMR (151 MHz; CD_3OD) δ 173.0, 170.1, 147.5, 146.1, 137.1, 133.4, 133.3, 125.1, 123.2, 123.1, 115.9, 114.9, 62.3, 58.1, 41.9, 30.7. *m/z* [ES+] 421 ([M+H]$^+$, 100%); *m/z* [HRMS ES-] found [M-H]$^-$ 419.0248. [$C_{18}H_{17}N_2O_5{}^{79}Br$-H]$^-$ requires 419.0233.

((3*S*)-1-(2-Bromophenyl)-6,7-dihydroxy-1,2,3,4-tetrahydroisoquinoline-3-carbonyl)glycine 3e

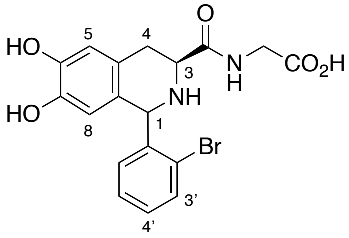

Compound 3e (both isomers) were prepared according to **procedure A** using L-Tyr-Gly (23.8 mg, 0.100 mmol) and 4-chlorobenzaldehyde (27.7 mg, 0.150 mmol). The product was purified by preparative HPLC (**Method 5,** Rt = 25.3 min) to give **3e** (the two diastereomers were assigned from the ^{1}H NMR NOE data) as a white solid (10 mg, 24% yield) (52% HPLC yield against product standards). M.p. 148 °C; ν_{max} (neat)/cm^{-1} 3066, 1666, 1189, 721; (1*S*,3*S*) (59% ratio): ^{1}H NMR (600 MHz; CD_3OD) δ 7.81-7.04 (4H, m, Ph-H), 6.73 (1H, s, 5-H), 6.23 (1H, s, 8-H), 6.15 (1H, s, 1-H), 4.20 (1H, dd, *J* = 11.7, 4.7 Hz, 3-H), 3.99 (1H, d, *J* = 17.8 Hz, NHC*H*H), 3.95 (1H, d, *J* = 17.8 Hz, NHCH*H*), 3.29 (1H, dd, *J* = 16.9, 4.7 Hz, 4-*H*H), 3.12 (1H, dd, *J* = 16.9, 11.7 Hz, 4-H*H*); ^{13}C NMR (151 MHz; CD_3OD) δ 172.9, 169.3, 147.5, 146.9, 137.5, 134.8, 130.0, 129.5, 126.8, 123.4, 121.2, 114.8, 114.7, 58.1, 51.8, 41.9, 30.6. (1*R*,3*S*) (41% ratio): ^{1}H NMR (600 MHz; CD_3OD) δ 7.81-7.04 (4H, m, Ph-H), 6.71 (1H, s, 5-H), 5.98 (1H, s, 8-H), 6.11 (1H, s, 1-H), 4.56 (1H, dd, *J* = 11.7, 4.7 Hz, 3-H), 4.05 (1H, d, *J* = 17.8 Hz, NHC*H*H), 4.00 (1H, d, *J* = 17.8 Hz, NHCH*H*), 3.40-3.29 (2H, m, 4-H_2); ^{13}C NMR (151 MHz; CD_3OD) δ 172.9, 169.3, 146.9, 146.2, 137.5, 134.8, 130.0, 129.5, 126.8, 123.5, 123.3, 115.5, 114.7, 61.6, 58.3, 41.9, 30.6. *m/z* [ES+] 421 ([M+H]$^+$, 100%); *m/z* [HRMS ES+] found [M+H]$^+$ 421.0393. [$C_{18}H_{17}N_2O_5{}^{79}Br$+H]$^+$ requires 421.0394.

((3*S*)-6,7-Dihydroxy-1-(4-methoxyphenyl)-1,2,3,4-tetrahydroisoquinoline-3-carbonyl)glycine 3g

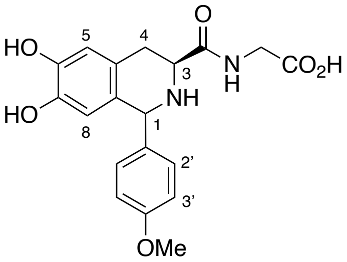

Compound 3g (both isomers) were prepared according to **procedure A** using L-Tyr-Gly (23.8 mg, 0.100 mmol) and 4-chlorobenzaldehyde (27.7 mg, 0.150 mmol). The product was purified by preparative HPLC (**Method 5,** Rt = 25.3 min) to give **3g** (the two diastereomers were assigned from the ^{1}H NMR NOE data) as a white solid (0.6 mg, 15% yield) (20% HPLC yield against product standards). M.p. 195 °C; ν_{max} (solution in CH_3OH)/cm^{-1} 3333, 2944, 1635, 1414; (1*R*,3*S*) (80% ratio): ^{1}H NMR (500 MHz; CD_3OD) *δ* 7.21 (2H, d, *J* = 8.8 Hz, 3'-H), 6.98 (2H, d, *J* = 8.8 Hz, 2'-H), 6.72 (1H, s, 5-H), 6.63 (1H, s, 8-H), 5.68 (1H, s, 1-H), 4.18 (1H, dd, *J* = 11.8, 5.3 Hz, 3-H), 4.02-3.90 (2H, m, NHC*H*$_2$), 3.81 (3H, s, OC*H*$_3$), 3.26 (1H, dd, *J* = 16.9, 5.3 Hz, 4-*H*H), 3.13 (1H, dd, *J* = 16.9, 11.8 Hz, 4-H*H*); ^{13}C NMR (126 MHz; CD_3OD) *δ* 170.6, 168.7, 160.8, 145.8, 145.7, 131.2, 128.3, 122.0, 121.2, 114.0, 113.9, 113.7, 57.6, 54.5, 50.3, 40.2, 28.8. (1*S*,3*S*) (20% ratio): ^{1}H NMR (500 MHz; CD_3OD) *δ* 7.39 (2H, d, *J* = 8.7 Hz, 3'-H), 7.03 (2H, d, *J* = 8.7 Hz, 2'-H), 6.69 (1H, s, 5-H), 6.14 (1H, s, 8-H), 5.54 (1H, s, 1-H), 4.41-4.39 (1H, m, 3-H), 4.00-3.97 (2H, m, NHC*H*$_2$), 3.85 (2H, s, OC*H*$_3$), 3.36-3.30 (2H, m, 4-H_2). ^{13}C NMR (176 MHz; CD_3OD) *δ* 173.3, 170.0, 163.4, 155.9, 144.7, 132.2, 131.5, 131.2, 123.0, 122.8, 114.5, 114.0, 61.0, 56.4, 47.7, 44.4, 28.9. *m/z* [ES+] 373 ([M+H]$^+$, 100%); *m/z* [HRMS ES+] found [M+H]$^+$ 373.1392. [$C_{19}H_{20}N_2O_6$+H]$^+$ requires 373.1394.

((3*S*)-1-(4-Formylphenyl)-6,7-dihydroxy-1,2,3,4-tetrahydroisoquinoline-3-carbonyl)glycine 3h

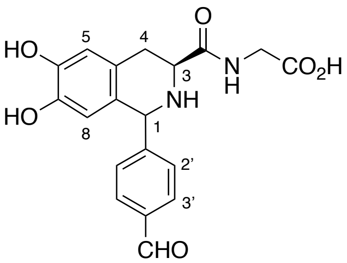

Compound 3h was prepared according to **procedure B** using L-Tyr-Gly (71.4 mg, 0.300 mmol) and terephthaldehyde (13.4 mg, 0.100 mmol). The product was purified by preparative HPLC (**Method 4**, R_t = 20.0 min) to give **3h** as a white solid (0.5 mg, 4% yield) (30% yield by HPLC against product standards). M.p. 150 °C; ν_{max} (neat)/cm^{-1} 3066, 1666, 1189; (1*R*,3*S*)

(major isomer): ^{1}H NMR (500 MHz; CD_3OD) δ 10.04 (1H, s, CHO), 7.99 (2H, d, J = 8.1 Hz, 3'-H), 7.52 (2H, d, J = 8.1 Hz, 2'-H), 6.76 (1H, s, 5-H), 6.36 (1H, s, 8-H), 5.85 (1H, s, 1-H), 4.19 (1H, dd, J = 11.6, 5.2 Hz, 3-H), 3.97-3.96 (2H, m, NHC*H₂*), 3.32-3.19 (2H, m, 4-H_2); ^{13}C (176 MHz; CD_3OD) δ 179.7, 178.5, 168.8, 148.3, 147.8, 137.6, 131,8, 129.7, 127.0, 123.0, 120.5, 114.0, 113.7, 57.6, 50.1, 40.3, 39.0. (1*S*,3*S*) (minor isomer): ^{1}H NMR (500 MHz; CD_3OD) δ 10.08 (1H, s, CHO), 8.05 (2H, d, J = 8.0 Hz, 3'-H), 7.70 (2H, d, J = 8.0 Hz, 2'-H), 6.73 (1H, s, 5-H), 6.35 (1H, s, 8-H), 5.74 (1H, s, 1-H), 4.47 (1H, dd, J = 11.1, 6.3 Hz, 3-H), 4.05-4.02 (2H, m, NHC*H_2*), 3.38-3.34 (2H, m, 4-H_2); ^{13}C (176 MHz; CD_3OD) δ 179.7, 178.5, 169.8, 150.8, 145.1, 135.2, 131,2, 130.9, 129.3, 123.0, 120.5, 114.5, 113.7, 52.8, 50.1, 40.9, 39.8. *m/z* [ES+] 371 ([M+H]$^+$, 100%); *m/z* [HRMS ES+] found [M+H]$^+$ 371.1237. [$C_{19}H_{18}N_2O_6$+H]$^+$ requires 371.1237.

3h-dimer *m/z* [ES+] 607 ([M+H]$^+$, 100%); *m/z* [HRMS ES+] found [M+H]$^+$ 607.2022. [$C_{30}H_{31}N_4O_{10}$+H]$^+$ requires 607.2035.

(3*S*,3'*S*)-1,1'-(1,4-Phenylene)bis(6,7-dihydroxy-1,2,3,4-tetrahydroisoquinoline-3-carboxylic acid) 3h-dimer

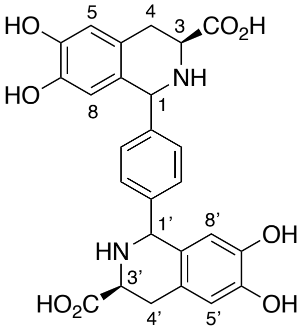

The dimer was prepared according to **Procedure B** using L-DOPA (80 mg, 0.40 mmol) and terephthaldehyde (26 mg, 0.20 mmol). The product was purified by preparative HPLC (**Method 4**, Rt = 13.7 min) to give **dimer products** (major product) as a white solid (3.1 mg, 4% yield) (40%, HPLC yield against product standards). M.p. 150 °C; ν_{max} (neat)/cm^{-1} 3066, 1666, 1189; (1*S*,1'*R*,3*S*,3'*S*) (50% ratio): ^{1}H NMR (700 MHz; CD_3OD) δ 7.55 (2H, d, J = 8.4 Hz, Ph-H), 7.47 (2H, d, J = 8.4 Hz, Ph-H), 6.75 (1H, s, 5'-H), 6.70 (1H, s, 5-H), 6.33 (1H, s, 8'-H), 6.04 (1H, s, 8-H), 5.84 (1H, s, 1'-H), 5.63 (1H, s, 1-H), 4.46 (1H, dd, J = 12.6, 5.1 Hz, 3-H), 4.20 (1H, dd, J = 10.1, 5.6 Hz, 3'-H), 3.40-3.22 (2H, m, 4'-H_2), 3.36-3.27 (2H, m, 4-H_2); ^{13}C NMR (176 MHz; CD_3OD) δ 171.1, 170.9, 147.1, 147.0, 146.9, 146.8, 139.6, 139.2, 132.1, 131.7, 123.6, 123.2, 123.0, 121.0, 115.7, 115.0, 114.8, 62.2, 58.7, 57.0, 52.1, 29.5, 29.1. (1*R*,1'*R*,3*S*,3'*S*) (50% ratio): ^{1}H NMR (700 MHz; CD_3OD) δ 7.40 (4H, s, Ph-H), 6.72 (2H, s, 5,5'-H), 6.29 (2H, s, 8,8'-H), 5.79 (2H, s, 1,1'-H), 4.16 (2H, dd, J = 10.1, 5.6 Hz, 3-H), 3.37-3.17 (4H, m, 4,4'-H_2); ^{13}C NMR (176 MHz; CD_3OD) δ 170.9, 147.2, 146.8, 139.4,132.0, 123.1,

122.9, 115.7, 114.8, 58.7, 51.9, 29.2. *m/z* [ES+] 493 ([M+H]$^+$, 100%); *m/z* [HRMS ES+] found [M+H]$^+$ 493.1602. [$C_{26}H_{24}N_2O_8$+H]$^+$ requires 493.1605.

((3*S*)-6,7-Dihydroxy-1-(pyren-1-yl)-1,2,3,4-tetrahydroisoquinoline-3-carbonyl)glycine 3i

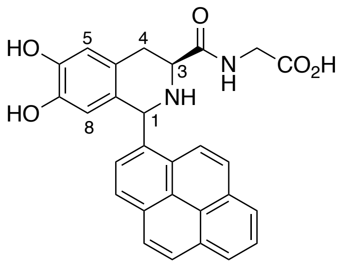

Compound 3i was prepared using L-DOPA-Gly (25.4 mg, 0.100 mmol, afforded from **2b** according to **Procedure D**), 1-pyrenecarbaldehyde (34.5 mg, 0.150 mmol) and sodium ascorbate (40 mg, 0.20 mmol) in 0.2 M KPi/MeOH/MeCN (1:1:1, 10 mL), at 50 °C, 18 h. The product was purified by preparative HPLC (**Method 4**, Rt = 12.6 min) to give **3i** as a white oil (5.0 mg, 10% yield) (50% HPLC yield against product standards). ν_{max} (solution in CH_3OH)/cm^{-1} 3332, 1630, 1418; ^{1}H NMR (500 MHz; CD_3OD) δ 8.62-7.68 (9H, m, Ph-H), 6.89 (1H, s, 5-H), 6.83 (1H, s, 8-H), 6.31-6.29 (1H, m, 1-H), 4.35-3.34 (1H, m, 3-H), 3.99 (1H, d, *J* = 18.0 Hz, NHC*H*H), 3.80 (1H, d, *J* = 18.0 Hz, NHCH*H*), 3.40-3.27 (2H, m, 4-H_2). ^{13}C NMR (176 MHz, CD_3OD) δ 176.0, 170.1, 146.7, 146.1, 138.5, 129.4, 129.1, 127.4, 126.5, 124.7, 121.8, 121.7, 121.6, 119.0, 118.0, 117.3, 117.2, 116.7, 116.6, 115.8, 113.7, 112.9, 67.7, 55.8, 42.6, 38.0. *m/z* [ES+] 467 ([M+H]$^+$, 100%); *m/z* [HRMS ES+] found [M+H]$^+$ 467.1598. [$C_{28}H_{22}N_2O_5$+H]$^+$ requires 467.1562.

4-(Pyren-1-yl)butanal 1j[5]

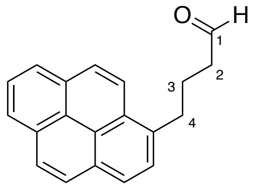

1-Pyrenebutyric acid (0.14 g, 0.50 mmol) and *N,O*-dimethylhydroxylamine hydrochloride (53.6 mg, 0.550 mmol) were used to synthesise a Weinreb amide precursor[6] according to **Procedure C**.[5] The Weinreb amide (83 mg, 0.25 mmol) was then dissolved in anhydrous THF (10 mL), cooled to 0 °C. $LiAlH_4$ (0.31 mL, 0.75 mmol) was added dropwise. The reaction was stirred at 0 °C for 30 min, then quenched with brine (10 mL), filtered, and concentrated *in vacuo*. The product was then extracted with EtOAc (3 x 10 mL), filtered, and concentrated *in vacuo* to give **1j** as a white solid (30 mg, 44%). M.p. 71-73 °C; R_f = 0.6 (hexane/ethyl acetate, 2:1). ^{1}H NMR (700 MHz; $CDCl_3$) δ 9.81 (1H, d, *J* = 1.5 Hz, C*H*O), 8.29-7.85 (9H, m, Ph-H), 3.42-3.38 (2H, m, 2-H_2), 2.59-2.75 (2H, m, 4-H_2), 2.23-2.19 (2H, m, 3-H_2); ^{13}C NMR (176 MHz,

$CDCl_3$) δ 202.3, 135.6, 131.5, 131.0, 130.1, 128.8, 127.6, 127.6, 127.4, 126.9, 126.0, 125.2, 125.1, 124.9, 123.3, 21.1, 14.3, 14.2. *m/z* [ES+] 273 ($[M+H]^+$, 100%). *m/z* [HRMS ES+] found $[M+H]^+$ 273.1265. $[C_{20}H_{16}O+H]^+$ requires 273.1273.

((3*S*)-6,7-Dihydroxy-1-(3-(pyren-2-yl)propyl)-1,2,3,4-tetrahydroisoquinoline-3-carbonyl)glycine 3j

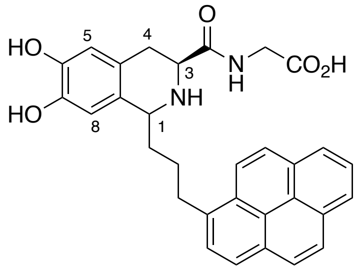

Compound 3j (both isomers) were prepared using L-DOPA-Gly (25.4 mg, 0.100 mmol, afforded form **2b** according to **Procedure D**), aldehyde (40.5 mg, 0.150 mmol) and sodium ascorbate (40 mg, 0.20 mmol) in 0.2 M KPi/MeOH/EtOAc (5:3:2, 10 mL), at 50 °C, 18 h. The product was purified by preparative HPLC (**Method 4**, Rt = 20.7 min) to give **3j** as a white solid (7.6 mg, 15% yield) (60% HPLC yield against product standards). M.p. 135-140 °C; ν_{max} (neat)/cm^{-1} 3066, 1666, 1189; (1*S*,3*S*) (50% ratio): ^1H NMR (500 MHz; CD_3OD) δ 8.38-7.93 (9H, m, Ph-H), 6.73 (1H, s, 5-H), 6.62 (1H, s, 8-H), 4.49-4.48 (1H, m, 1-H), 4.09 (1H, dd, *J* = 11.9, 5.1 Hz, 3-H), 4.01-3.98 (2H, m, NHC*H₂*), 3.55-3.53 (2H, m, C*H₂*Ph), 3.47-3.45 (2H, m, CHC*H₂*), 3.17-3.13 (2H, m, 4-H_2), 2.15-2.10 (2H, m, $CH_2H_2CH_2$); ^{13}C NMR (126 MHz; CD_3OD) δ 168.5, 168.0, 145.3, 144.6, 131.4, 130.6, 130.3, 128.8, 127.2, 127.1, 126.7, 125.6, 124.5, 124.4, 121.6, 120.2, 114.7, 113.0, 56.1, 55.1, 54.9, 34.0, 32.7, 27.8, 27.7. (1*R*,3*S*) (50% ratio): ^1H NMR (500 MHz; CD_3OD) δ 8.38-7.93 (9H, m, Ph-H), 6.62 (1H, s, 5-H), 6.61 (1H, s, 8-H), 4.44-4.42 (1H, m, 1-H), 4.32 (1H, dd, *J* = 11.3, 5.8 Hz, 3-H), 4.02 (1H, d, *J* = 17.7 Hz, NHC*H*H), 3.96 (1H, d, *J* = 17.7 Hz, NHCH*H*), 3.53-3.52 (2H, m, C*H₂*Ph), 3.47-3.44 (2H, m, CHC*H₂*), 3.22 (1H, d, *J* = 16.8, 5.8 Hz, 4-*H*H), 3.01 (1H, d, *J* = 16.8, 11.3 Hz, 4-H*H*), 2.13-2.11 (2H, m, $CH_2H_2CH_2$); ^{13}C NMR (126 MHz; CD_3OD) δ 172.2, 169.4, 145.5, 144.6, 131.4, 130.6, 130.3, 128.8, 127.2, 127.1, 126.7, 125.6, 124.5, 124.4, 121.6, 120.2, 114.7, 113.0, 61.8, 54.9, 50.6, 34.0, 32.6, 27.7, 27.6. *m/z* [ES+] 509 ($[M+H]^+$, 100%); *m/z* [HRMS ES+] found $[M+H]^+$ 509.2606. $[C_{24}H_{37}N_4O_8+H]^+$ requires 509.2606.

Leu-enkephalin 2c[2]

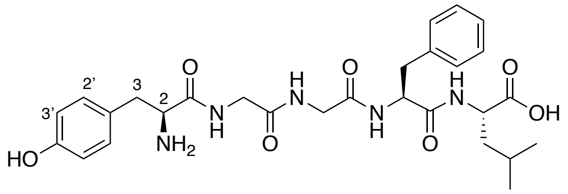

The protected peptide was synthesized according to **Procedure C** using amino acids (4 mmol) as shown in the scheme below. The product was purified by silica gel column chromatography (chloroform/MeOH, 100%/0% to 90%/10%) to give the **protected peptide precursor** as a brown oil (1.6 g, 60%).

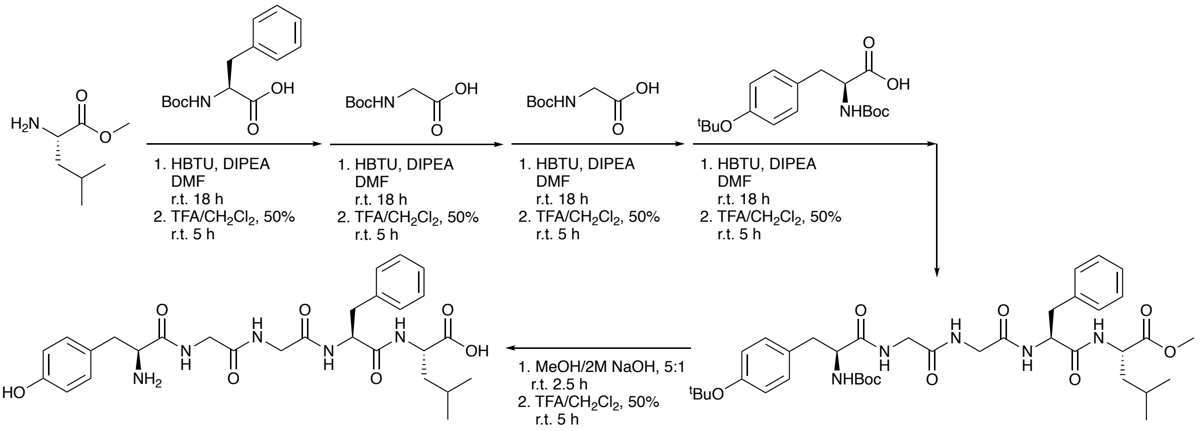

The protected peptide precursor (0.290 g, 0.40 mmol) was then dissolved in 12 mL of a 5:1 mixture of MeOH/2 M NaOH, and the resulting solution was stirred at room temperature for 2.5 h. The product was concentrated and then diluted into 10 mL of water and washed with ether (3 × 10 mL). The aqueous layer was acidified to pH 2 using 1 M HCl and extracted by EtOAc (3 × 10 mL), and the organic layer was concentrated *in vacuo*. The resulting compound was dissolved in CH_2Cl_2 (5 mL), cooled to 0 °C, and TFA (5 mL) was added. The reaction was stirred at room temperature for 5 h and then concentrated to give the crude product. The crude product was purified by preparative HPLC (**Method 4,** Rt = 17.0 min) to give **2c** as a yellow solid (227 mg, 80% yield). M.p. 136 °C; $[\alpha]_D^{26}$ -48.7 (c 0.3, CH_3OH); ν_{max} (neat)/cm^{-1} 3335, 3296, 1633; ^{1}H NMR (600 MHz; CD_3OD) δ 7.29-7.18 (5H, m, Ph-H), 7.13 (2H, d, *J* = 8.5 Hz, 3'-H), 6.81 (2H, d, *J* = 8.5 Hz, 2'-H), 4.73 (1H, dd, *J* = 9.6, 4.7 Hz, C*H*CH_2Ph), 4.43 (1H, dd, *J* = 8.0, 6.9 Hz, C*H*$CH_2CH(CH_3)_2$), 4.09 (1H, dd, *J* = 8.3, 6.2 Hz, 2-H), 3.98 (1H, d, *J* = 16.3 Hz, NHC*H*H), 3.80 (1H, d, *J* = 16.3 Hz, NHCH*H*), 3.91 (1H, d, *J* = 16.9 Hz, NHC*H*H), 3.76 (1H, d, *J* = 16.9 Hz, NHCH*H*), 3.20-3.17 (2H, m, 3-*H*H, CHC*H*HPh), 2.99-2.98 (2H, m, 3-H*H*, CHCH*H*Ph), 1.74-1.72 (1H, m, $CH_2C\mathit{H}(CH_3)_2$), 1.67-1.65 (2H, m, $C\mathit{H_2}CH(CH_3)_2$), 0.97 (3H, d, *J* = 6.3 Hz, $CH_2CHC\mathit{H_3}(CH_3)$), 0.92 (3H, d, *J* = 6.3 Hz, $CH_2CHCH_3(C\mathit{H_3})$); ^{13}C NMR (176 MHz; CD_3OD) δ 175.6, 173.5, 171.3, 170.9, 170.9, 158.2, 138.2, 131.4, 130.3, 129.3, 129.3, 127.6, 125.8, 116.7, 56.0, 55.5, 52.2, 43.8, 43.0, 41.4, 38.8, 37.5, 25.8, 23.2, 21.7. *m/z* [ES+] 556 ([M+H]$^+$, 100%); *m/z* [HRMS ES+] found [M+H]$^+$ 556.2770. $[C_{28}H_{37}N_5O_7+H]^+$ requires 556.2766.

((3*S*)-1-Benzyl-6,7-dihydroxy-1,2,3,4-tetrahydroisoquinoline-3-carbonyl)glycylglycyl-L-phenylalanyl-L-leucine 4a

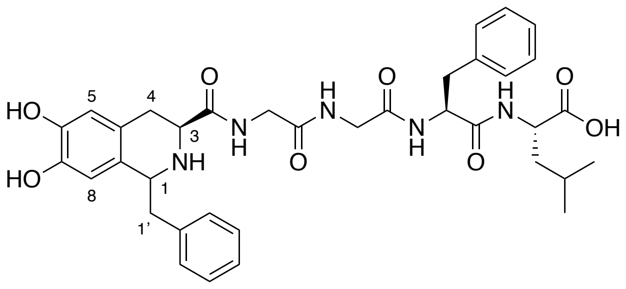

Compound 4a (both isomers) was prepared according to **procedure A** using **2c** (55.5 mg, 0.100 mmol) and phenylacetaldehyde (17.5 μL, 0.150 mmol). The product was purified by preparative HPLC (**Method 5,** Rt = 31.0 min) to give **4a** as a white solid (0.3 mg, 4.5% yield) (40% HPLC yield against product standards). M.p. 154 °C; ν_{max} (neat)/cm^{-1} 3336, 3314, 1706, 1620; ^{1}H NMR (700 MHz; CD_3OD) *δ* 7.40-7.26 (10H, m, Ph-H), 6.81 (1H, s, 5-H), 6.66 (1H, s, 8-H), 4.76 (1H, dd, *J* = 8.7, 5.5 Hz, C*H*CH_2Ph), 4.68 (1H, dd, *J* = 9.5, 4.8 Hz, 1-H), 4.41 (1H, t, *J* = 7.4 Hz, C*H*$CH_2CH(CH_3)_2$), 4.08 (1H, dd, *J* = 11.3, 5.9 Hz, 3-H), 3.97 (1H, d, *J* = 16.3 Hz, NHC*H*H), 3.87 (1H, d, *J* = 16.3 Hz, NHCH*H*), 3.76 (1H, d, *J* = 17.2 Hz, NHC*H*H), 3.71 (1H, d, *J* = 17.2 Hz, NHCH*H*), 3.58 (1H, dd, *J* = 14.6, 5.5 Hz, CHC*H*HPh), 3.20-3.10 (4H, m, CHCH*H*Ph, 4-H_2, 1'-*H*H), 2.87 (1H, dd, *J* = 14.0, 9.5 Hz, 1'-H*H*), 1.73-1.63 (1H, m, C*H*$(CH_3)_2$), 1.66-1.60 (2H, m, CHCH_2CH$(CH_3)_2$), 0.94 (3H, d, *J* = 6.2 Hz, CHC$H_3$$(CH_3)$), 0.89 (3H, d, *J* = 6.2 Hz, $CHCH_3$(CH_3)); ^{13}C NMR (176 MHz; CD_3OD) *δ* 175.6, 173.4, 171.0, 170.9, 146.8, 146.6, 138.6, 138.1, 137.0, 136.7, 130.4, 130.2, 129.7, 128.8, 123.4, 122.9, 116.1, 113.5, 57.4, 55.5, 52.3, 47.8, 41.5, 43.9, 43.4, 40.7, 38.5, 30.4, 25.8, 23.1, 22.0. *m/z* [ES+] 674 ([M+H]$^+$, 100%); *m/z* [HRMS ES+] found [M+H]$^+$ 674.3186. $[C_{36}H_{43}N_5O_8+H]^+$ requires 674.3184.

((3*S*)-6,7-Dihydroxy-1-phenyl-1,2,3,4-tetrahydroisoquinoline-3-carbonyl)glycylglycyl-L-phenylalanyl-L-leucine 4b

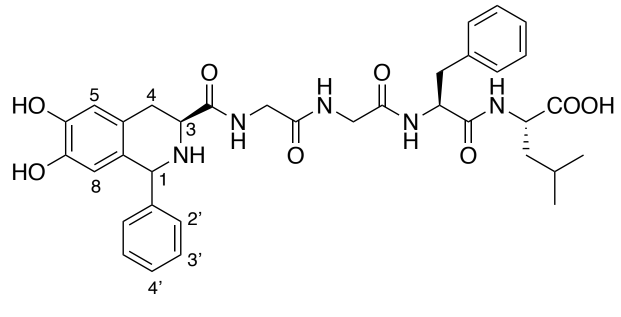

Compound 4b (both isomers) was prepared according to **procedure A** using **2c** (55.5 mg, 0.100 mmol) and benzaldehyde (15.3 μL, 0.150 mmol). The product was purified by preparative HPLC (**Method 5,** Rt = 30.9 min) to give **4b** as a white solid (1.1 mg, 20% yield) (50% HPLC yield against product standards). M.p. 130 °C; ν_{max} (neat)/cm^{-1} 3311, 1707, 1637; (1*R*,3*S*) (70% ratio): ^{1}H NMR (700 MHz; CD_3OD) *δ* 7.27-7.24 (10H, m, Ph-H), 6.70 (1H, s, 5-H), 6.11 (1H, s, 8-H), 5.64 (1H, s, 1-H), 4.65 (1H, dd, *J* = 9.3, 4.0 Hz, C*H*CH_2Ph), 4.43 (1H, t,

J = 7.4 Hz, C*H*CH$_2$CH(CH$_3$)$_2$), 4.27 (1H, dd, *J* = 11.7, 5.3 Hz, 3-H), 3.96 (1H, d, *J* = 16.3 Hz, NHC*H*H), 3.89 (1H, d, *J* = 17.0 Hz, NHC*H*H), 3.81 (1H, d, *J* = 16.3 Hz, NHCH*H*), 3.62 (1H, d, *J* = 17.0 Hz, NHCH*H*), 3.36-3.32 (1H, m, 4-*H*H), 3.20-3.14 (2H, m, CHC*H*HPh, 4-H*H*), 2.84 (1H, dd, *J* = 14.0, 9.3 Hz, CHCH*H*Ph), 1.75-1.65 (1H, m, CH$_2$C*H*(CH$_3$)$_2$), 1.68-1.58 (2H, m, CHC*H$_2$*CH(CH$_3$)$_2$), 0.95 (3H, d, *J* = 6.4 Hz, CHC*H$_3$*(CH$_3$)), 0.91 (3H, d, *J* = 6.4 Hz, CHCH$_3$(C*H$_3$*)); ^{13}C NMR (176 MHz; CD$_3$OD) *δ* 175.5, 175.4, 173.5, 171.0, 170.6, 147.6, 146.4, 138.3, 137.4, 131.5, 131.3, 130.3, 129.7, 128.4, 127.7, 122.5, 121.1, 115.5, 114.7, 59.6, 55.7, 52.2, 51.6, 43.9, 42.7, 41.5, 38.6, 30.2, 25.6, 23.2, 21.7. (1*S*,3*S*) (30% ratio): ^{1}H NMR (700 MHz; CD$_3$OD) *δ* 7.27-7.24 (10H, m, Ph-H), 6.70 (1H, s, 5-H), 6.11 (1H, s, 8-H), 5.64 (1H, s, 1-H), 4.70 (1H, dd, *J* = 9.8, 4.6 Hz, C*H*CH$_2$Ph), 4.47-4.45 (1H, m, 3-H), 4.44-4.42 (1H, m, C*H*CH$_2$CH(CH$_3$)$_2$), 4.06 (1H, d, *J* = 16.3 Hz, NHC*H*H), 4.00 (1H, d, *J* = 17.0 Hz, NHC*H*H), 3.96 (1H, d, *J* = 16.3 Hz, NHCH*H*), 3.89 (1H, d, *J* = 17.0 Hz, NHCH*H*), 3.38-3.30 (1H, m, 4-*H*H), 3.19-3.15 (1H, m, CHC*H*HPh), 2.97-2.92 (2H, m, 4-H*H*, CHCH*H*Ph), 1.72-1.68 (1H, m, CH$_2$C*H*(CH$_3$)$_2$), 1.65-1.60 (2H, m, CHC*H$_2$*CH(CH$_3$)$_2$), 0.95 (3H, d, *J* = 6.4 Hz, CHC*H$_3$*(CH$_3$)), 0.91 (3H, d, *J* = 6.4 Hz, CHCH$_3$(C*H$_3$*)); ^{13}C NMR (176 MHz; CD$_3$OD) *δ* 175.9, 175.5, 173.5, 171.2, 170.9, 146.3, 146.2, 138.4, 131.4, 130.9, 130.7, 130.4, 130.3, 129.7, 128.4, 127.6, 123.9, 123.5, 115.5, 115.3, 63.2, 55.8, 55.7, 52.1, 44.0, 41.5, 41.3, 38.2, 30.2, 25.8, 23.2, 21.7. *m/z* [ES+] 660 ([M+H]$^+$, 100%); *m/z* [HRMS ES+] found [M+H]$^+$ 660.3029. [C$_{35}$H$_{41}$N$_5$O$_8$+H]$^+$ requires 660.3028.

((3S)-1-(4-Chlorophenyl)-6,7-dihydroxy-1,2,3,4-tetrahydroisoquinoline-3-carbonyl)glycylglycyl-L-phenylalanyl-L-leucine 4c

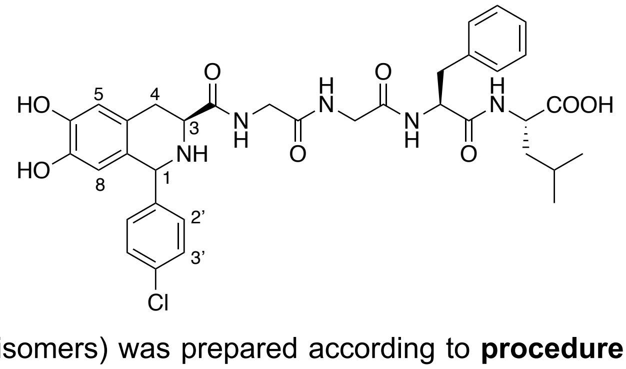

Compound 4c (both isomers) was prepared according to **procedure A** using **2c** (55.5 mg, 0.100 mmol) and 4-bromobenzaldehyde (23.8 mg, 0.150 mmol). The product was purified by preparative HPLC (**Method 5,** Rt = 32.4 min) to give **4c** as a white solid (0.7 mg, 10% yield) (25% HPLC yield against product standards). M.p. 142 °C; ν_{max} (neat)/cm^{-1} 3326, 3277, 1639, 987; (1*R*,3*S*) (70% ratio): ^{1}H NMR (700 MHz; CD$_3$OD) *δ* 7.46 (2H, d, *J* = 8.5 Hz, 3'-H), 7.33 (2H, d, *J* = 8.5 Hz, 2'-H), 7.26-7.12 (5H, m, Ph-H), 6.73 (1H, s, 5-H), 6.37 (1H, s, 8-H), 5.75 (1H, s, 1-H), 4.70 (1H, dd, *J* = 9.8, 4.5 Hz, C*H*CH$_2$Ph), 4.40 (1H, t, *J* = 7.4 Hz, C*H*CH$_2$CH(CH$_3$)$_2$), 4.21 (1H, dd, *J* = 11.7, 5.2 Hz, 3-H), 3.96 (1H, d, *J* = 16.3 Hz, NHC*H*H),

3.89 (1H, d, *J* = 17.0 Hz, NHC*H*H), 3.81 (1H, d, *J* = 16.3 Hz, NHCH*H*), 3.62 (1H, d, *J* = 17.0 Hz, NHCH*H*), 3.34-3.30 (1H, m, 4-*H*H), 3.20-3.14 (2H, m, CHC*H*HPh, 4-H*H*), 2.99 (1H, dd, *J* = 14.0, 9.8 Hz, CHCH*H*Ph), 1.71-1.69 (1H, m, $CH_2C\mathit{H}(CH_3)_2$), 1.66-1.60 (2H, m, $CHC\mathit{H_2}CH(CH_3)_2$), 0.95 (3H, d, *J* = 6.2 Hz, $CHC\mathit{H_3}(CH_3)$), 0.91 (3H, d, *J* = 6.2 Hz, $CHCH_3(C\mathit{H_3})$); ^{13}C NMR (176 MHz; CD_3OD) *δ* 175.5, 173.3, 171.4, 171.3, 171.0, 147.3, 146.8, 138.3, 136.6, 133.3, 133.2, 130.3, 129.5, 122.1, 121.5, 115.6, 114.8, 58.8, 55.4, 52.2, 51.9, 43.6, 42.9, 41.3, 38.7, 30.3, 25.7, 23.3, 22.1. (1*S*,3*S*) (30% ratio): ^{1}H NMR (700 MHz; CD_3OD) *δ* 7.50 (2H, d, *J* = 8.4 Hz, 3'-H), 7.45 (d, *J* = 8.4 Hz, 2'-H), 7.26-7.12 (5H, m, Ph-H), 6.70 (1H, s, 5-H), 6.09 (1H, s, 8-H), 5.65 (1H, s, 1-H), 4.67 (1H, dd, *J* = 8.6, 6.0 Hz, $C\mathit{H}CH_2Ph$), 4.43 (1H, dd, *J* = 11.6, 5.5 Hz, 3-H), 4.36 (1H, dd, *J* = 8.8, 5.9 Hz, $C\mathit{H}CH_2CH(CH_3)_2$), 3.98 (1H, d, *J* = 16.3 Hz, NHC*H*H), 3.88 (1H, d, *J* = 16.3 Hz, NHCH*H*), 3.86 (1H, d, *J* = 16.8 Hz, NHC*H*H), 3.79 (1H, d, *J* = 16.8 Hz, NHCH*H*), 3.33-3.31 (2H, m, $4\text{-}H_2$), 3.09 (1H, dd, *J* = 13.9, 6.0 Hz, CHC*H*HPh), 2.87 (1H, dd, *J* = 13.9, 8.6 Hz, CHCH*H*Ph), 1.71-1.69 (1H, m, $CH_2C\mathit{H}(CH_3)_2$), 1.62-1.58 (2H, m, $CHC\mathit{H_2}CH(CH_3)_2$), 0.93 (3H, d, *J* = 6.2 Hz, $CHC\mathit{H_3}(CH_3)$), 0.89 (3H, d, *J* = 6.2 Hz, $CHCH_3(C\mathit{H_3})$); ^{13}C NMR (176 MHz; CD_3OD) *δ* 175.5, 174.4, 173.8, 171.1, 170.8, 146.8, 146.7, 138.0, 136.8, 136.7, 133.6, 133.1, 132.7, 130.5, 130.3, 129.8, 127.7, 123.6, 123.5, 116.3, 115.1, 62.3, 58.2, 55.5, 52.3, 44.1, 42.9, 41.5, 39.0, 30.3, 25.7, 23.2, 21.7. *m/z* [ES+] 694 ([M+H]$^+$, 100%); *m/z* [HRMS ES+] found [M+H]$^+$ 694.2632. $[C_{35}H_{40}N_5O_8{}^{35}Cl+H]^+$ requires 694.2638.

((3*S*)-1-(4-Bromophenyl)-6,7-dihydroxy-1,2,3,4-tetrahydroisoquinoline-3-carbonyl)glycylglycyl-L-phenylalanyl-L-leucine 4d

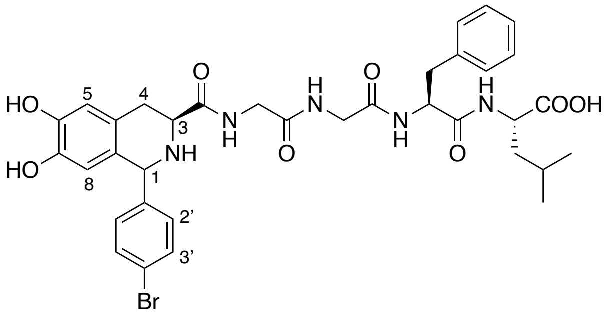

Compound 4d (both isomers) was prepared according to **procedure A** using **2c** (55.5 mg, 0.100 mmol) and 4-bromobenzaldehyde (27.7 mg, 0.150 mmol). The product was purified by preparative HPLC (**Method 5,** Rt = 32.9 min) to give **4d** as a white solid (1.4 mg, 20% yield) (36% HPLC yield against product standards). M.p. 165 °C; ν_{max} (neat)/cm^{-1} 3325, 1638, 987; (1*R*,3*S*) (83% ratio): ^{1}H NMR (700 MHz; CD_3OD) *δ* 7.62 (2H, d, *J* = 8.3 Hz, 3'-H), 7.26 (2H, d, *J* = 8.3 Hz, 2'-H), 7.26-7.11 (5H, m, Ph-H), 6.73 (1H, s, 5-H), 6.37 (1H, s, 8-H), 5.75 (1H, s, 1-H), 4.70 (1H, dd, *J* = 9.9, 4.6 Hz, $C\mathit{H}CH_2Ph$), 4.40 (1H, t, *J* = 7.3 Hz, $C\mathit{H}CH_2CH(CH_3)_2$), 4.22 (1H, dd, *J* = 11.8, 5.2 Hz, 3-H), 3.96 (1H, d, *J* = 16.3 Hz, NHC*H*H), 3.88 (1H, d, *J* = 17.0 Hz,

NHC*H*H), 3.82 (1H, d, *J* = 16.3 Hz, NHCH*H*), 3.64 (1H, d, *J* = 17.0 Hz, NHCH*H*), 3.33-3.31 (1H, m, 4-*H*H), 3.16-3.13 (2H, m, 4-H*H,* CHC*H*HPh), 2.99 (1H, dd, *J* = 14.0, 9.9 Hz, CHCH*H*Ph), 1.69-1.67 (1H, m, $CH_2CH(CH_3)_2$) 1.66-1.61 (2H, m, $CHCH_2CH(CH_3)_2$), 0.96 (3H, d, *J* = 6.2 Hz, $CHCH_3(CH_3)$), 0.88 (3H, d, *J* = 6.2 Hz, $CHCH_3(CH_3)$); ^{13}C NMR (176 MHz; CD_3OD) δ 175.4, 173.7, 171.3, 171.1, 170.7, 147.0, 146.9, 138.2, 138.1, 136.8, 133.5, 133.4, 133.3, 133.2, 130.2, 129.7, 127.6, 122.7, 121.4, 115.5, 114.8, 58.8, 55.3, 52.3, 51.7, 43.7, 42.7, 41.3, 38.7, 30.1, 25.7, 23.3, 21.7. (1*S*,3*S*) (17% ratio): ^{1}H NMR (700 MHz; CD_3OD) δ 7.66 (2H, d, *J* = 8.3 Hz, 3'-H), 7.39 (2H, d, *J* = 8.3 Hz, 2'-H), 7.26-7.11 (5H, m, Ph-H), 6.71 (1H, s, 5-H), 6.09 (1H, s, 8-H), 5.67 (1H, s, 1-H), 4.68 (1H, dd, *J* = 8.7, 6.3 Hz, C*H*CH_2Ph), 4.46 (1H, dd, *J* = 11.9, 5.5 Hz, 3-H), 4.37 (1H, dd, *J* = 8.8, 5.9 Hz, C*H*$CH_2CH(CH_3)_2$), 4.00 (1H, d, *J* = 16.3 Hz, NHC*H*H), 3.89 (1H, d, *J* = 16.3 Hz, NHCH*H*), 3.85 (1H, d, *J* = 17.0 Hz, NHC*H*H), 3.81 (1H, d, *J* = 17.0 Hz, NHCH*H*), 3.33-3.31 (2H, m, 4-H_2), 3.07 (1H, dd, *J* = 13.9, 6.3 Hz, CHC*H*HPh), 2.85 (1H, dd, *J* = 13.9, 8.7 Hz, CHCH*H*Ph), 1.69-1.67 (1H, m, $CH_2CH(CH_3)_2$) 1.66-1.64 (2H, m, $CHCH_2CH(CH_3)_2$), 0.93 (3H, d, *J* = 6.2 Hz, $CHCH_3(CH_3)$), 0.89 (3H, d, *J* = 6.2 Hz, $CHCH_3(CH_3)$); ^{13}C NMR (176 MHz; CD_3OD) δ 176.1, 175.4, 173.7, 171.2, 171.1, 146.5, 146.4, 138.2, 138.1, 136.8, 133.5, 133.4, 133.3, 133.2, 130.2, 129.7, 127.6, 123.9, 122.9, 115.5, 114.8, 62.4, 57.8, 55.4, 52.2, 43.9, 43.5, 41.3, 38.7, 30.2, 25.7, 23.3, 21.7. *m/z* [ES+] 738 ([M+H]$^+$, 100%); *m/z* [HRMS ES+] found [M+H]$^+$ 738.2133. $[C_{35}H_{40}N_5O_8{}^{79}Br+H]^+$ requires 738.2128.

((3S)-1-(2-Bromophenyl)-6,7-dihydroxy-1,2,3,4-tetrahydroisoquinoline-3-carbonyl)glycylglycyl-L-phenylalanyl-L-leucine 4e

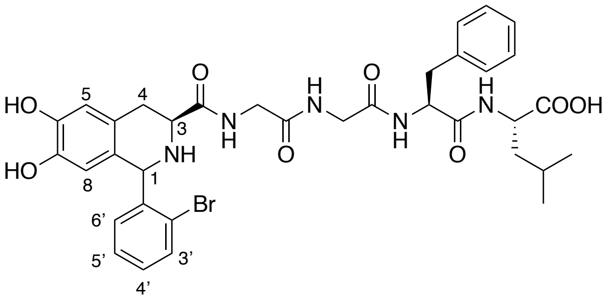

Compound 4e (both isomers) was prepared according to **procedure A** using **2c** (55.5 mg, 0.100 mmol) and 4-bromobenzaldehyde (27.7 mg, 0.150 mmol). The product was purified by preparative HPLC (**Method 5,** Rt = 31.4 min) to give **4e** as a white solid (1.5 mg, 20% yield) (77% HPLC yield against product standards). M.p. 150 °C; ν_{max} (neat)/cm^{-1} 3334, 3294, 1639, 987; (1*R*,3*S*) (50% ratio): ^{1}H NMR (700 MHz; CD_3OD) δ 7.26-7.16 (9H, m, Ph-H), 6.73 (1H, s, 5-H), 6.23 (1H, s, 8-H), 6.15 (1H, s, 1-H), 4.68-4.64 (1H, m, C*H*CH_2Ph), 4.43 (1H, t, *J* = 7.4 Hz, C*H*$CH_2CH(CH_3)_2$), 4.23 (1H, dd, *J* = 11.5, 4.8 Hz, 3-H), 4.02-3.73 (4H, m, NHCH_2, NHCH_2), 3.40-3.36 (1H, m, 4-*H*H), 3.15-3.11 (2H, m, 4-H*H*, CHC*H*HPh), 2.94-2.90 (1H, m, CHCH*H*Ph),

1.71-1.69 (1H, m, $CH_2CH(CH_3)_2$), 1.66-1.63 (2H, m, $CHCH_2CH(CH_3)_2$), 0.95 (3H, d, J = 6.2 Hz, $CHCH_3(CH_3)$), 0.86 (3H, d, J = 6.2 Hz, $CHCH_3(CH_3)$); ^{13}C NMR (176 MHz; CD_3OD) δ 173.9, 172.3, 171.7, 169.8, 146.1, 145.4, 137.0, 135.6, 132.3, 128.7, 128.3, 126.3, 126.2, 122.0, 119.8, 114.1, 113.2, 57.3, 54.2, 50.8, 50.6, 42.3, 41.8, 40.1, 37.3, 29.0, 24.5, 21.8, 20.3. (1*R*,3*S*) (50% ratio): ^1H NMR (700 MHz; CD_3OD) δ 7.26-7.16 (9H, m, Ph-H), 6.70 (1H, s, 5-H), 6.14 (1H, s, 1-H), 6.00 (1H, s, 8-H), 4.68-4.64 (1H, m, $CHCH_2Ph$), 4.58 (1H, dd, J = 10.7, 6.9 Hz, 3-H), 4.32 (1H, dd, J = 9.8, 4.5 Hz, $CHCH_2CH(CH_3)_2$), 4.02-3.73 (4H, m, $NHCH_2$, $NHCH_2$), 3.40-3.36 (2H, m, 4-H_2), 3.15-3.11 (1H, m, CHC*H*HPh), 2.84 (1H, dd, J = 14.0, 9.4 Hz, CHCH*H*Ph), 1.72-1.70 (1H, m, $CH_2CH(CH_3)_2$), 1.65-1.62 (2H, m, $CHCH_2CH(CH_3)_2$), 0.95-0.90 (6H, m, $CH(CH_3)_2$); ^{13}C NMR (176 MHz; CD_3OD) δ 173.9, 172.3, 171.7, 169.8, 146.1, 145.4, 137.0, 135.6, 132.3, 128.7, 128.3, 126.3, 126.2, 122.0, 119.8, 114.5, 113.2, 60.6, 57.3, 54.2, 50.8, 42.3, 41.8, 40.1, 37.3, 29.0, 24.5, 20.3. *m/z* [ES+] 738 ($[M+H]^+$, 100%); *m/z* [HRMS ES+] found $[M+H]^+$ 738.2128. $[C_{35}H_{40}N_5O_8{}^{79}Br+H]^+$ requires 738.2133.

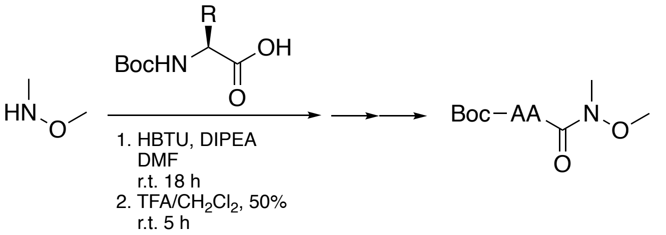

N,O-Dimethylhydroxylamine hydrochloride (97 mg, 1.0 mmol) and amino acids (1.1 mmol) were used to prepare Boc-amino acid Weinreb amides according to **Procedure C**.

tert-Butyl ((_S_)-3-methyl-1-oxo-1-(((_S_)-1-oxopropan-2-yl)amino)butan-2-yl)carbamate 5a

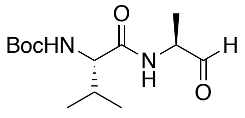

The corresponding Weinreb amide (66.2 mg, 0.200 mmol) was dissolved in anhydrous THF (10 mL), cooled to 0 °C. $LiAlH_4$ (0.3 mL, 0.7 mmol) was added dropwise. The reaction was stirred at 0 °C for 30 min, then quenched with brine (10 mL), filtered, and concentrated *in vacuo*. The product was then extracted with EtOAc (3 x 10 mL), filtered, and concentrated *in vacuo* to give **5a** (21.8 mg, 50%) as a pale-yellow oil. R_f = 0.3 (hexane/ethyl acetate, 1:1); ν_{max} (neat)/cm^{-1} 3332, 1630, 1418; ^1H NMR (600 MHz; $CDCl_3$) δ 9.54 (1H, d, J = 3.3 Hz, CHO), 5.01 (1H, m, $CHCH(CH_3)_2$), 3.96 (1H, m, $CHCH_3$), 2.16 (1H, m, $CHCH(CH_3)_2$), 1.44 (9H, s, Boc-H), 1.37 (3H, d, J = 7.4 Hz, $CHCH_3$), 0.97 (3H, d, J = 6.8 Hz, $CHCH_3(CH_3)$), 0.92 (3H, d, J = 6.8 Hz, $CHCH_3(CH_3)$); ^{13}C NMR (176 MHz; $CDCl_3$) δ 174.1, 173.2, 157.3, 81.0, 61.6, 57.2,

31.9, 28.7, 19.6, 18.3, 14.5. *m/z* [ES+] 273 ($[M+H]^+$, 100%); *m/z* [HRMS ES+] found $[M+H]^+$ 273.1807. $[C_{13}H_{24}N_2O_4+H]^+$ requires 273.1808.

tert-Butyl ((_S_)-1-oxo-1-(((S)-1-oxopropan-2-yl)amino)-3-phenylpropan-2-yl)carbamate 5b

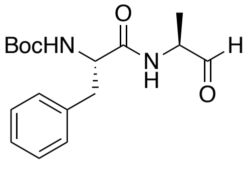

The corresponding Weinreb amide (prepared according to **Procedure C**, 38 mg, 0.10 mmol) was added in anhydrous THF (5 mL) and cooled to 0 °C under argon. Lithium aluminium hydride (0.1 mL, 0.2 mmol) was added drop wise, and the reaction was stirred at 0 °C for 30 min. The reaction was quenched with the addition of brine (5 mL), filtered, and concentrated *in vacuo*, resuspended in H_2O (5 mL). The aqueous layer was extracted with EtOAc (3 x 10 mL), and the organic layer was concentrated to give **5b** as a yellow oil (25 mg, 80%). Rf = 0.3 (hexane/ethyl acetate, 1:1); IR ν_{max} (solution in CH_3OH)/cm^{-1} 2944, 1442; ^{1}H NMR (700 MHz; $CDCl_3$) δ 9.41 (1H, s, CHO), 7.30-7.25 (5H, m, Ph-H), 4.40-3.37 (1H, m, C*H*CH_3), 4.39-3.38 (1H, m, C*H*CH_2Ph), 3.12 (1H, dd, *J* = 14.0, 6.8 Hz, CHC*H*HPh), 3.04 (1H, dd, *J* = 14.0, 6.8 Hz, CHCH*H*Ph), 1.42 (9H, s, Boc-H), 1.29 (3H, d, *J* = 7.5 Hz, CHC*H*$_3$); ^{13}C NMR (176 MHz, CD_3OD) δ 209.8, 173.5, 172.8, 138.6, 130.3, 130.3, 129.3, 80.5, 57.4, 50.3, 39.2, 28.6, 14.4. *m/z* [ES+] 321 ($[M+H]^+$, 100%). *m/z* [HRMS ES+] found $[M+H]^+$ 321.1804. $[C_{17}H_{24}N_2O_4+H]^+$ requires 321.1808.

tert-Butyl (1-(((_S_)-3-methyl-1-oxo-1-(((_S_)-1-oxopropan-2-yl)amino)butan-2-yl)amino)-1-oxo-3-phenylpropan-2-yl)carbamate 5c

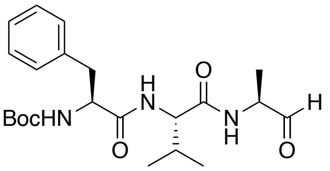

The corresponding Weinreb amide (prepared according to **Procedure C**, 53 mg, 0.10 mmol) was added in anhydrous THF (5 mL) and cooled to 0 °C under argon. Lithium aluminium hydride (0.17 mL, 0.30 mmol) was added drop wise, and the reaction was stirred at 0 °C for 30 min. The reaction was quenched with the addition of brine (5 mL), filtered, and concentrated *in vacuo*, resuspended in H_2O (5 mL). The aqueous layer was extracted with EtOAc (3 x 10 mL), and the organic layer was concentrated to give **5c** as a colorless oil (12 mg, 30%). Rf = 0.5 (hexane/ethyl acetate, 1:2); ν_{max} (solution in CH_3OH)/cm^{-1} 3692, 1650; ^{1}H NMR (500 MHz; CD_3OD) δ 9.44 (1H, s, CHO), 7.26-7.22 (5H, m, Ph-H), 4.35 (1H, dd, *J* = 9.4, 5.1 Hz,

C*H*CH$_2$Ph), 4.18-4.16 (1H, m, C*H*CH(CH$_3$)$_2$), 3.91 (1H, d, *J* = 6.7 Hz, C*H*CH$_3$), 3.12 (1H, dd, *J* = 13.9, 5.1 Hz, CHC*H*HPh), 2.81 (1H, dd, *J* = 13.9, 9.4 Hz, CHCH*H*Ph), 2.05-2.02 (1H, m, CHC*H*(CH$_3$)$_2$), 1.36 (9H, s, Boc-H), 1.13 (3H, s, CHC*H$_3$*), 0.99-0.95 (6H, m, CH(C*H$_3$*)$_2$). ^{13}C NMR (176 MHz, CD$_3$OD) *δ* 182.9, 175.5, 172.7, 159.6, 138.5, 130.3, 129.5, 127.6, 80.5, 59.8, 57.3, 50.5, 38.8, 32.3, 28.4, 19.5, 14.4. *m/z* [ES+] 420 ([M+H]$^+$, 100%); *m/z* [HRMS ES+] found [M+H]$^+$ 420.2480. [$C_{22}H_{33}N_3O_5$+H]$^+$ requires 420.2493.

((3*S*)-1-((*S*)-1-((*S*)-2-((*tert*-Butoxycarbonyl)amino)-3-methylbutanamido)ethyl)-6,7-dihydroxy-1,2,3,4-tetrahydroisoquinoline-3-carbonyl)glycine 6a

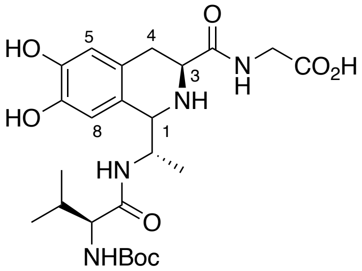

Compound 6a (both isomers) were prepared using L-DOPA-Gly (25.4 mg, 0.100 mmol, afforded from **2b** according to **Procedure D**), Boc-Val-Ala-H (37.5 mg, 0.150 mmol) and sodium ascorbate (40 mg, 0.20 mmol) in 0.2 M KPi/DMSO (1:1, 10 mL), at 50 °C, 24 h. The product was purified by preparative HPLC (**Method 4**, Rt = 20.0 min) to give **6a** as a white solid (5 mg, 9% yield) (48% HPLC yield against product standards). M.p. 126 °C; ν_{max} (neat)/cm^{-1} 3066, 1666, 1189, 721; ^{1}H NMR (700 MHz; CD$_3$OD) δ 6.65 (1H,s, 5-H), 6.64 (1H, s, 8-H), 4.66 (1H, dd, *J* = 9.3, 6.5 Hz, 3-H), 4.33 (1H, d, *J* = 8.8 Hz, 1-H), 4.29-2.26 (1H, m, C*H*CH$_3$), 4.07 (1H, d, *J* = 17.6 Hz, NHC*H*H), 3.89 (1H, d, *J* = 17.6 Hz, NHCH*H*), 3.75 (1H, d, *J* = 5.9 Hz, C*H*CH(CH$_3$)$_2$), 3.35 (1H, dd, *J* = 17.1, 6.5 Hz, 4-*H*H), 3.07 (1H, dd, *J* = 17.1, 9.3 Hz, 4-H*H*), 2.08-2.04 (1H, m, CHC*H*(CH$_3$)$_2$), 1.47 (9H, s, Boc-H), 1.31 (3H, d, *J* = 6.9 Hz, CHC*H$_3$*), 0.95 (3H, d, *J* = 6.8 Hz, CHC*H$_3$*(CH$_3$)), 0.93 (d, *J* = 6.8 Hz, 3H, CHCH$_3$(C*H$_3$*)). ^{13}C NMR (176 MHz; CD$_3$OD) *δ* 176.3, 175.6, 171.4, 168.5, 147.1, 146.2, 122.5, 120.3, 116.4, 116.1, 81.8, 62.5, 61.0, 52.9, 48.6, 41.8, 31.2, 30.9, 28.6, 19.2, 17.7. *m/z* [ES+] 509 ([M+H]$^+$, 100%); *m/z* [HRMS ES+] found [M+H]$^+$ 509.2606. [$C_{24}H_{37}N_4O_8$+H]$^+$ requires 509.2606.

((3*S*)-1-((*S*)-1-((*S*)-2-((*tert*-butoxycarbonyl)amino)-3-phenylpropanamido)ethyl)-6,7-dihydroxy-1,2,3,4-tetrahydroisoquinoline-3-carbonyl)glycine 6b

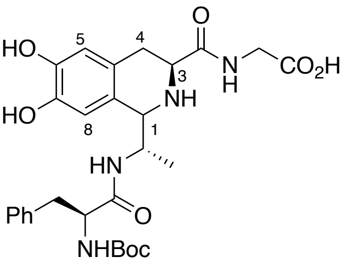

Compound 6b was prepared using L-DOPA-Gly-OH (25.4 mg, 0.100 mmol, afforded form **2b** according to **Procedure D**), Boc-Phe-Ala-H (26 mg, 0.10 mmol) and sodium ascorbate (40 mg, 0.20 mmol) in 0.2 M KPi/MeOH/EtOAc (5:3:2, 10 mL), at 50 °C, 18 h. The product was purified by preparative HPLC (**Method 4**, Rt = 22.7 min) to give **6b** as a white solid (5 mg, 8% yield) (35% HPLC yield against product standards). M.p. 154 °C; ν_{max} (solution in CH_3OH)/cm^{-1} 3324, 2944, 1650, 1412; ^1H NMR (700 MHz; CD_3OD) δ 7.28-7.17 (5H, m, Ph-H), 6.62 (1H, s, 5-H), 6.59 (1H, s, 8-H), 4.58-4.55 (1H, m, 3-H), 4.22 (1H, d, *J* = 8.9 Hz, 1-H), 4.10-4.09 (1H, m, C*H*CH_3), 4.08-4.07 (1H, m, C*H*CH_2Ph), 4.01-3.92 (2H, m, NHCH_2), 3.07-2.89 (2H, m, 4-H_2), 2.54 (2H, d, *J* = 8.5 Hz, CHCH_2Ph), 1.39 (9H, s, Boc-H), 1.18 (3H, d, *J* = 7.0 Hz, CHCH_3); ^{13}C NMR (176 MHz; CD_3OD) *δ* 175.7, 175.6, 171.6, 170.1, 146.3, 146.2, 130.1, 129.4, 129.5, 127.6, 120.4, 120.3, 116.1, 116.0, 80.8, 60.9, 58.0, 52.9, 48.7, 41.8, 38.3, 30.9, 28.4, 17.3. *m/z* [ES+] 557 ($[M+H]^+$, 100%); *m/z* [HRMS ES+] found $[M+H]^+$ 557.2610. $[C_{28}H_{37}N_4O_8+H]^+$ requires 557.2606.

((3*S*)-1-((*S*)-1-((*S*)-2-((*S*)-2-((tert-Butoxycarbonyl)amino)-3-phenylpropanamido)-3-methylbutanamido)ethyl)-6,7-dihydroxy-1,2,3,4-tetrahydroisoquinoline-3-carbonyl)glycine 6c

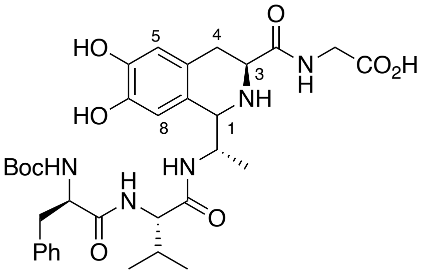

Compound 6c was prepared using L-DOPA-Gly-OH (25.4 mg, 0.100 mmol, afforded form **2b** according to **Procedure D**), Boc-Phe-Val-Ala-H (42 mg, 0.10 mmol) and sodium ascorbate (40 mg, 0.20 mmol) in 0.2 M KPi/MeOH/EtOAc (5:3:2, 10 mL), at 50 °C, 18 h. The product was purified by preparative HPLC (**Method 4**, Rt = 21.9 min) to give **6c** as a white solid (6 mg, 9% yield) (35% HPLC yield against product standards). M.p. 165 °C; ν_{max} (solution in CH_3OH)/cm^{-1} 3002, 2944, 1650; ^1H NMR (500 MHz; CD_3OD) δ 7.29-6.96 (5H, m, Ph-H), 6.91-6.60 (2H, m, 5-H, 8-H), 4.72-4.63 (1H, m, C*H*CH_2Ph), 4.54-4.43 (1H, m, 1-H), 4.35-4.28 (1H, m, 3-H), 4.26-4.19 (1H, m, C*H*CH_3), 4.07-3.99 (1H, m, C*H*$CH(CH_3)_2$), 3.83-3.78 (2H, m,

NHC*H*$_2$), 3.33-3.08 (2H, m, CHC*H*$_2$Ph), 3.13-2.85 (2H, m, 4-H_2), 2.09-2.03 (1H, m, C*H*(CH_3)$_2$), 1.40-1.35 (12H, m, Boc-H, CHC*H*$_3$), 0.98-0.89 (6H, m, CH(C*H*$_3$)$_2$); ^{13}C NMR (126 MHz, CD_3OD) δ 178.0, 173.4, 171,7, 164.6, 150.5, 145.5, 145.4, 137.6, 128.7, 128.6, 126.3, 123.0, 119.4, 114.9, 114.1, 79.3, 60.5, 59.1, 56.6, 56.3, 51.8, 48.7, 37.2, 37.1, 29.8, 27.2, 24.8, 17.8, 15.6. *m/z* [ES+] 656 ([M+H]$^+$, 100%); *m/z* [HRMS ES+] found [M+H]$^+$ 656.3291. [$C_{33}H_{45}N_5O_9$+H]$^+$ requires 656.3301.

6. Molecular Docking Figures

Substrates for docking were energy minimized using ChemDraw Professional 16.0 (PerkinElmer). AutoDockTools (Scripps) was used to define rotatable bonds in ligands and to prepare pdbqt files for docking. Docking was performed using AutoDock Vina (Scripps). Docking of wild-type *Cn*TYR was performed using the crystal structure of *Bm*TYR (PDB code: 3NPY) as a template. A box size of (50, 54, 46) was used with a box centre of (21.4, 15.4, 9.4).

Protein structures and docking results were visualised using UCSF Chimera 1.15rc.

One of the possible models was shown in Figure S1.

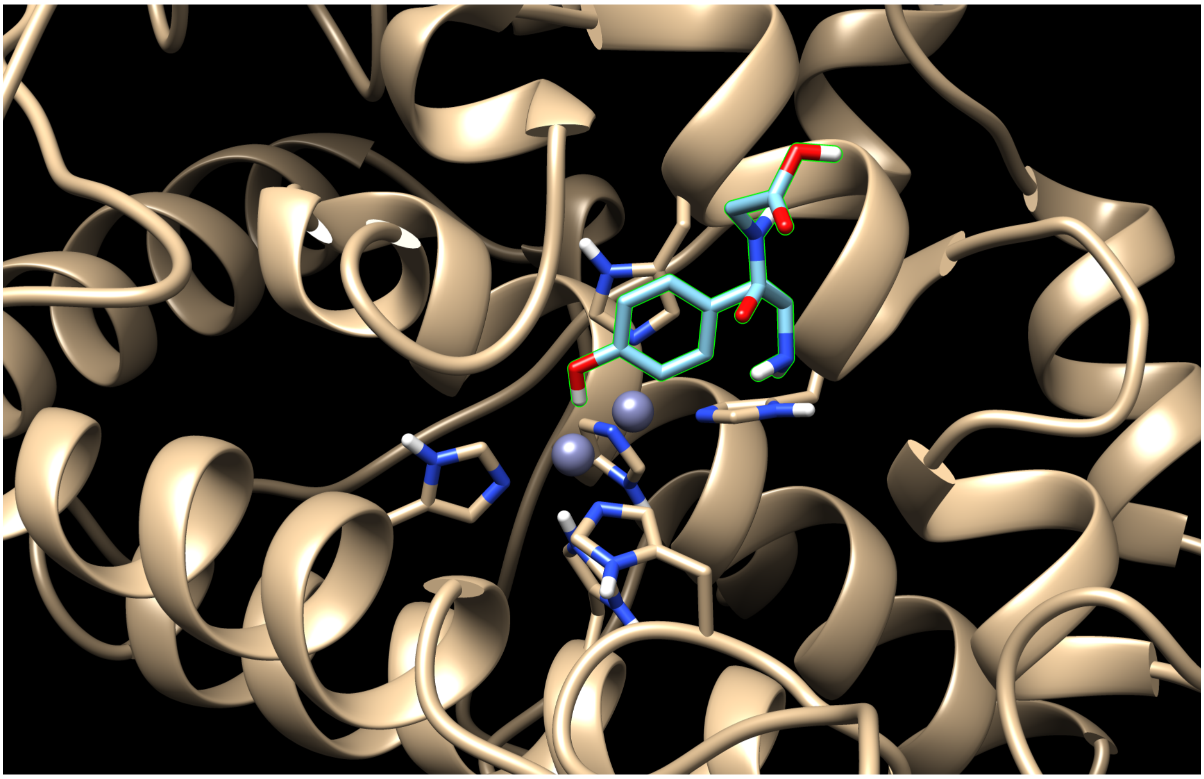

Figure S1. Molecular docking of dipeptide **2b** in a productive conformation with *Cn*TYR using Autodock Viva.

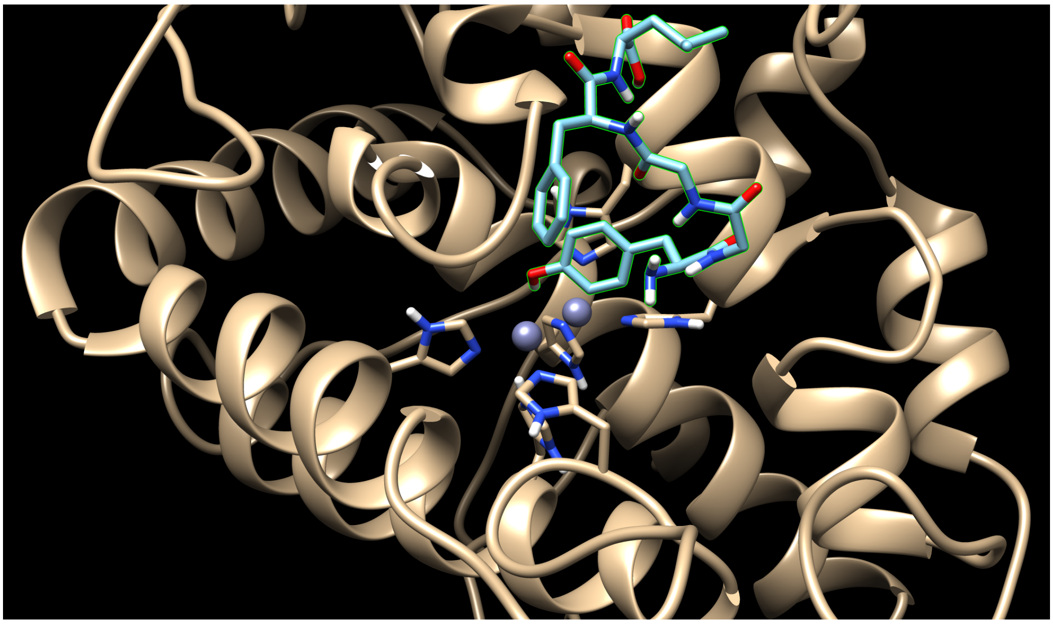

Figure S2. Molecular docking of pentapeptide **2c** in a productive conformation with *Cn*TYR using Autodock Viva.

7. Abbreviations

DIPEA	*N,N*-Diisopropylethylamine
DMF	Dimethylformamide
DMSO	Dimethyl sulfoxide
DOPA	3,4-Dihydroxyphenylalanine
EDC	*N*-(3-Dimethylaminopropyl)-*N'*-ethylcarbodiimide hydrochloride
EtOAc	Ethyl acetate
HBTU	*O*-(Benzotriazol-1-yl)-*N,N,N',N'*-tetramethyluronium hexafluorophosphate
HEPES	4-(2-hydroxyethyl)-1-piperazineethanesulfonic acid
HOBT	1-Hydroxybenzotriazol hydrate
HPLC	High-performance liquid chromatography
LCMS	Liquid chromatography–mass spectrometry
Mp	Melting point
NMR	Nuclear magnetic resonance
PSR	Pictet-Spengler reaction
TFA	Trifluoroacetic acid
THF	Tetrahydrofuran
TLC	Thin layer chromatography
TYR	Tyrosinase
UV	Ultraviolet

8. References

1. Y. Wang, N. Tappertzhofen, D. Méndez-Sánchez, M. Bawn, B. Lyu, J. M. Ward and H. C. Hailes, *Angew. Chem., Int. Ed.,* 2019, **58**, 10120-10125.
2. M. N. Moller, D. M. Hatch, H. Y. Kim and N. A. Porter, *J. Am. Chem. Soc.*, 2012, **134**, 16773-16780.
3. G. Giorgioni, F. Claudi, S. Ruggieri, M. Ricciutelli, G. F. Palmieri, A. Di Stefano, P. Sozio, L. S. Cerasa, A. Chiavaroli, C. Ferrante, G. Orlando and R. A. Glennon, *Bioorg. Med. Chem.*, 2010, **18**, 1834-1843.
4. O. Arnaud, A. Koubeissi, L. Ettouati, R. Terreux, G. Alame, C. Grenot, C. Dumontet, A. Di Pietro, J. Paris and P. Falson, *J. Med. Chem.*, 2010, **53**, 6720-6729.
5. D. E. Scott, M. D. Aloisio, J. F. Rodriguez, M. Morimoto, R. J. Hamilton, O. Brown, R. R. Tykwinski and J. M. Stryker, *Adv. Synth. Catal.,* 2021, **363**, 4720-4727.
6. D. J. Lippincott, P. J. Trejo-Soto, F. Gallou and B. H. Lipshutz, *Org. Lett.*, 2018, **20**, 5094-5097.
